# Implicit sub-stepping scheme for critical state soil models

**DOI:** 10.1007/s00366-026-02309-1

**Published:** 2026-04-01

**Authors:** Hoang-Giang Bui, Jelena Niníc, Günther Meschke

**Affiliations:** 1 Department of Engineering, Durham University, Durham, UK; 2 Institute for Structural Mechanics, Ruhr University Bochum, Bochum, Germany; 3 School of Engineering, University of Birmingham, Birmingham, UK

**Keywords:** Modified Cam-Clay, CASM, Sub-stepping scheme, Implicit integration

## Abstract

The stress integration of critical soil model is usually based on implicit Euler algorithm, where the stress predictor is corrected by employing a return mapping algorithm. In the case of a large load step, the solution of the local nonlinear system to compute the plastic multiplier may not be attained. To overcome this problem, a sub-stepping scheme is generally used to improve the convergence of the local nonlinear system solution strategy. Nevertheless, the complexity of the tangent operator of the sub-stepping scheme is high. This complicates the use of Newton–Raphson algorithm to obtain global quadratic convergence. In this paper, a formulation for consistent tangent operator is developed for implicit sub-stepping integration for the modified Cam-Clay model and unified Clay and Sand model. This formulation is highly efficient and can be used with problem involving arbitrary large load step, such as tunnel simulation.

## Introduction

The introduction of critical soil models marks an important advance in computational geomechanics. Based on elastoplastic theory, the model is first proposed by [22, 25] as Cam-Clay model (CC), and subsequently improved as modified Cam-Clay model [21]. The modified Cam-Clay model (MCC) is demonstrated as effective to predict main attributes of the constitutive behavior of normally consolidated soft cohesive soils [12], yet with some deficiencies for modelling of highly overconsolidated soils. Later, Yu [33] generalized the standard Cam-Clay model to allow modeling the behavior of sand. The unified model, denoted as CASM [33, 34], has the ability to resolve some drawbacks of the standard Cam-Clay models, such as the overestimation of the strength on the “dry side” or the behavior of loose sand in undrained tests, while limiting the number of parameters to seven, which is an advantage to adopt the model both in academic research and practical applications.

To apply the critical soil models in numerical simulations, an integration algorithm is required. In the context of implicit methods, the backward Euler algorithm is typically used. This is frequently resorted to the return mapping algorithm [27, 29], which ensures quadratic convergence of the Newton–Raphson algorithm if certain conditions are satisfied. The advantage of the implicit method is its robustness, second-order accuracy and able to use with large load step. Nevertheless, the implicit algorithm typically requires the high-order derivatives of yield functions and plastic flow rule. The return mapping algorithm has been successfully applied to Cam-Clay models in [2] and, being extended to the finite strain regime, in [28]. Further applications of the return map algorithm to extensions of the Cam Clay model have been suggested in [4, 6, 7].

In contrast to implicit methods, explicit methods, based on the forward Euler integration, requires only the first order approximation of the yield surface and plastic flow rule and it is considered to be more straightforward to implement. Because only first order accuracy is attained, the load step must be sufficiently small to reduce the accumulated error. The efficiency and robustness of explicit algorithms has been substantially improved by using sub-stepping algorithms [26, 30, 31, 35]. An adaptive error control for sub-stepping algorithm is proposed in [1].

It is well known from the analysis of convergence properties of stability of the Newton algorithm, from applications reported in the literature [5, 10], and also experienced by the authors, that the computational robustness of implicit integration schemes applied to elastoplastic models, and, in particular, to critical state models, is limited, and that no convergence of the local iterative scheme at the integration point level is attained when the load step is large. Therefore, it is crucial to sub-divide the load into smaller increments to ensure the existence of local admissible stress state and the convergence of the local iteration, to compute the internal variables and the plastic increment. Because the sub-load increment can be adjusted, the sub-stepping scheme is highly effective for problems, in which the loading path is difficult to control. An example of this class of problems are excavation problems, which are a priori stress control problems if no arc length methods are applied. A consistent sub-stepping scheme has been proposed for elastoplastic models in [19]. Nevertheless, the trial stress predictor uses a linear extrapolation based on a (constant) elastic tangent, which is typically used for elastoplastic material models. Therefore, this scheme is not optimal for critical state soil models, because the stress–strain rule in the elastic region of the critical state model is nonlinear [21].

The convergence difficulty of the single-step algorithm can be solved by reducing the global load step. However, in geotechnical application, especially the excavation problem, it is hard to control since the element is deactivated sequentially. To be able to control the load step, it is needed to introduce artificial external force at the excavation boundary to resist the reaction force and reduce it gradually, i.e., stress relaxation approach. This process is cumbersome to implement and prone to error. On the other hand, the alternative is to employ the explicit algorithm with error control [1]. By combining with initial stiffness scheme, the convergence rate is typically linear.

To address the above shortcomings of the conventional solutions and to improve the robustness and accuracy of the integration of the constitutive law, and to develop the consistent tangent in order to maintain the global quadratic convergence rate via implicit Newton–Raphson scheme, we propose in Sect. 2 of this paper, a sub-stepping algorithm for the implicit integration which is designed specifically for the critical state soil models, in particular the Modified Cam Clay model and the Clay and Sand model. The automatic integration procedure makes use of the more accurate nonlinear approximation of hydrostatic pressure. The accuracy of the implicit sub-stepping scheme is validated and its effectiveness is verified through selected numerical examples presented in Sect. 3. The findings from these analyses are concluded in Sect. 4.

## Implicit integration of the critical state soil models

In this section, the basic governing equations of the Modified Cam-Clay (MCC) (Sect. 2.1) model and the Clay and Soil model (Sect. 2.2) are summarized before an implicit integration algorithm incorporating sub-stepping is elaborated for both models in subsections Sect. 2.4.

### Brief summary of the modified Cam-Clay model

The Modified Cam-Clay (MCC) model [23, 25] is characterized by a yield surface formulated in the *p*-*q* plane by:

1 $$\begin{aligned} f^{\text {MCC}}(p, q, p_c) = \left( \dfrac{q}{M} \right) ^2 + p (p - p_c), \end{aligned}$$

by the associative plastic potential $g^{\text {MCC}}(p, q, p_c) = f^{\text {MCC}}(p, q, p_c)$ and by the nonlinear stress–strain relation in the elastic domain:

2 $$\begin{aligned} \dot{p} = K \dot{\varepsilon }^e_v \,, \qquad \dot{\textbf{s}} = 2 \mu \dot{\boldsymbol{\varepsilon }}^e_d. \end{aligned}$$

The shear modulus and the pressure-dependent compression modulus *K* are given as

3 $$\begin{aligned} K = \dfrac{1 + e}{\kappa } p \,, \qquad \mu =Kr \,, \qquad r = \dfrac{3}{2} \dfrac{1-2\nu }{1+\nu }, \end{aligned}$$

and the hardening rule is specified as

4 $$\begin{aligned} \dot{p}_c = \theta p_c \dot{\varepsilon }^p_v \,, \qquad \theta = \dfrac{1+e}{\lambda -\kappa }. \end{aligned}$$

The plastic flow rule reads

5 $$\begin{aligned} \dot{\boldsymbol{\varepsilon }}^p = \dot{\theta } \left( \dfrac{1}{3} \dfrac{\partial g^{\text {MCC}}}{\partial p} \textbf{I} \right) + \sqrt{\dfrac{3}{2}} \dfrac{\partial g^{\text {MCC}}}{\partial q} \hat{\textbf{n}} \,, \quad \hat{\textbf{n}} = \dfrac{\textbf{s}}{\Vert \textbf{s} \Vert } \end{aligned}$$

In Eqs. (1) to (4), $M, e, \lambda , \kappa , \nu$ are material constants and have following meanings: *M* is the slope of the critical state line, *e* is the void ratio, $\lambda$ is the compression index, $\kappa$ is the swelling index and $\nu$ is the Poisson ratio. For the details on the material parameters and their roles in reflecting the characteristics of soil, the reader is referred to [32]. $p=\dfrac{1}{3} \text {tr} (\boldsymbol{\sigma })$ denotes the hydrostatic pressure, *q* the deviatoric invariant, $q=\sqrt{3/2 \, \textbf{s}:\textbf{s}}$, with $\textbf{s}=\boldsymbol{\sigma } - p \textbf{I}$ as the deviatoric stress, and $p_c$ the preconsolidation pressure. The subscript $(\bullet )_v$ denotes the volumetric component and $(\bullet )_d$ the deviatoric component of the strain. The superscript $(\bullet )^e$ denotes the elastic component and $(\bullet )^p$ denotes the plastic component of the strain. The flow rule (5) is applicable for other critical state model as well providing suitable plastic potential function. The void ratio *e* is considered not to change significantly during a load step and is kept fixed within one sub-step, i.e., increment. In this work, we don’t consider the dependency of *M* on the Lode angle.

### Brief summary of the CASM model

The Clay and Sand model (CASM) model, as proposed in [33], proposes the yield surface:

6 $$\begin{aligned} f^{\text {CASM}}(p, q, p_c) = \left( \dfrac{q}{Mp} \right) ^N + \dfrac{1}{\ln R} \ln \dfrac{p}{p_c}, \end{aligned}$$

and the non-associative plastic potential, which adopts Rowe’s stress dilatancy relation [24]:

7 $$\begin{aligned} g^{\text {CASM}}(p, q) &= 3M \ln \dfrac{p}{\beta } \\&\quad+ (3+2M) \ln \left( 2 \dfrac{q}{p} + 3 \right)\\&\quad - (3-M) \ln \left( 3 - \dfrac{q}{p} \right) . \end{aligned}$$

$\beta$ is a size parameter and dependent on the stress state. It can be obtained by solving $g^{\text {CASM}}(p, q) = 0$ at a plastic state. The stress–strain relation in the elastic domain and the hardening rule (4) are similar to the MCC model (see (2) and (3)). The flow rule is analogous to Eq. (5) of MCC but using the plastic potential (7), hence the non-associativity is assumed by default. In addition to the material parameters *M*, *e*, $\lambda$, $\kappa$, $\mu$, the CASM model is characterized by additional parameters *N* and *R* defining the shape of the yield surface in the deviatoric plane. *N* is the positive parameter controlling the shape of the yield surface and is typically range from 1.5 to 2 to match with the MCC yield criterion [33]. *R* is the spacing ratio of the soil particles. The CASM model can be adjusted to reproduce the standard Cam-Clay model. For example, the original Cam-Clay model can be obtained by setting $N=1$ and $\ln R=1$, while the MCC model can be approximated by setting $R=2$ and a suitable value for *N*. For more details on the theoretical basis of CASM model, the reader is referred to [33].

### One-step implicit integration scheme

In the one-step integration scheme, the hydrostatic pressure is approximated by integrating the rate form of nonlinear stress–strain relation (see Eq. 2) [3]. It is summarized here to serve as the basis for sub-stepping scheme proposed in the next Subsection.

#### Stress integration algorithm

We denote $\{p_{n+1}, q_{n+1}, (p_c)_{n+1}\}$ as the state variables at the current load step and $\Delta \boldsymbol{\varepsilon } = \boldsymbol{\varepsilon }_{n+1} - \boldsymbol{\varepsilon }_n$ as the incremental total strain obtained from the solution of the equilibrium equations on the structural level for load step $n+1$. For brevity, the step index $n+1$ of the current step is neglected in what follows. In the predictor step, which assumes pure elastic loading ($\Delta \varepsilon ^e_v = \Delta \epsilon _v$), the stresses and shear stiffness are calculated as:

8 $$\begin{aligned} p &= p_n \, \text {exp} \left( \dfrac{1+e}{\kappa } \Delta \epsilon _v \right) \,, \, \\\textbf{s} &= \textbf{s}_n + 2 \bar{\mu } \Delta \boldsymbol{\varepsilon }_d \,, \\\, p_c &= (p_c)_n \,, \, \bar{\mu } = \dfrac{p - p_n}{\Delta \epsilon _v} r. \end{aligned}$$

If the yield condition in the predictor step is violated, i.e., $f(p, q, p_c) > 0$, the material point is subjected to plastic loading. Subsequently, $\{p,q,p_c\}$ along with the increment of the plastic multiplier $\Delta \phi$ need to be determined from the solution of the following set of nonlinear equations, which holds for any Critical State model, see Appendix  A:

9 $$\begin{aligned} h_1&= p - p_n \, \text {exp} \left( \dfrac{1+e}{\kappa } \Delta \varepsilon ^e_v \right) = 0, \end{aligned}$$

10 $$\begin{aligned} h_2&= q - \sqrt{\dfrac{3}{2}} \Vert \textbf{s}_n + 2\bar{\mu } \Delta \boldsymbol{\varepsilon }_d^e \Vert = 0 \, , \quad \bar{\mu } = \dfrac{p - p_n}{\Delta \epsilon _v^e} r, \end{aligned}$$

11 $$\begin{aligned} h_3&= p_c - \left( p_c \right) _n \text {exp} \left( \theta \Delta \epsilon _v^p \right) = 0, \end{aligned}$$

12 $$\begin{aligned} h_4&= f(p, q, p_c) = 0 . \end{aligned}$$

The solution for the set of unknowns $\{ p, q, p_c, \Delta \phi \}$ of the nonlinear system Eqs. (9) to (12) can be determined by using the implicit Newton–Raphson algorithm. For details of the linearization of the residuals from the nonlinear system when using the Newton–Raphson method for the MCC model, the reader is referred to [3]. For the CASM model, the reader is referred to Appendix  D.

The void ratio *e* is initialized a priori as

13 $$\begin{aligned} e = e_0 - \lambda \log p_c - \kappa \log \dfrac{p}{p_c} \end{aligned}$$

in which $e_0$ is the initial void ratio. This is to keep the void ratio compatible with the stress on the critical state line [25]. Moreover, the void ratio is also updated using Eq. (13) after the load step is complete. This holds for both one-step and multi-step scheme in Sect. 2.4.

#### Consistent tangent operator

With the solution for the stress state and the hardening variables at hand, the consistent tangent operator can be defined as:

14 $$\begin{aligned} {\mathbb {C}} = \dfrac{\partial \boldsymbol{\sigma } }{\partial \Delta \boldsymbol{\varepsilon }} = \dfrac{\partial \textbf{s}}{\partial \Delta \boldsymbol{\varepsilon }} + \textbf{I} \otimes \dfrac{\partial p}{\partial \Delta \boldsymbol{\varepsilon }}. \end{aligned}$$

The consistent tangent operator ${\mathbb {C}}$ is crucial to preserve the quadratic rate of convergence of the fully implicit Newton–Raphson strategy. In the elastic state, the components $\dfrac{\partial \textbf{s}}{\partial \Delta \boldsymbol{\varepsilon }}$ and $\dfrac{\partial p}{\partial \Delta \boldsymbol{\varepsilon }}$ of (14) can be straightforwardly calculated by taking the linearization of (8):

15 $$\begin{aligned} &\dfrac{\partial p}{\partial \Delta \boldsymbol{\varepsilon }} = K \textbf{I} \,,\\& \quad \dfrac{\partial \textbf{s}}{\partial \Delta \boldsymbol{\varepsilon }} = 2 \bar{\mu } {\mathbb {I}}_d \\&+ 2 \Delta \boldsymbol{\varepsilon }_d \otimes \dfrac{\partial \bar{\mu }}{\partial \Delta \boldsymbol{\varepsilon }} \,,\\& \quad \dfrac{\partial \bar{\mu }}{\partial \Delta \boldsymbol{\varepsilon }} = \dfrac{K r - \bar{\mu }}{\Delta \epsilon _v} \textbf{I} \,,\\& \quad K = \dfrac{1+e}{\kappa } p. \end{aligned}$$

In the case that the strain increment in the integration point is leading to an elasto-plastic response in the current increment, $\dfrac{\partial p}{\partial \Delta \boldsymbol{\varepsilon }}$, $\dfrac{\partial p_c}{\partial \Delta \boldsymbol{\varepsilon }}$ and $\dfrac{\partial \textbf{s}}{\partial \Delta \boldsymbol{\varepsilon }}$ can be written in terms of $\dfrac{\partial \Delta \phi }{\partial \Delta \boldsymbol{\varepsilon }}$ as:

16 $$\begin{aligned} \dfrac{\partial p}{\partial \Delta \boldsymbol{\varepsilon }} &= \textbf{A} + a \dfrac{\partial \Delta \phi }{\partial \Delta \boldsymbol{\varepsilon }} \,,\\ \qquad \dfrac{\partial p_c}{\partial \Delta \boldsymbol{\varepsilon }} &= \textbf{B} + b \dfrac{\partial \Delta \phi }{\partial \Delta \boldsymbol{\varepsilon }} \,,\\ \qquad \dfrac{\partial \textbf{s}}{\partial \Delta \boldsymbol{\varepsilon }} &= {\mathbb {D}} + \textbf{D} \otimes \dfrac{\partial \Delta \phi }{\partial \Delta \boldsymbol{\varepsilon }}. \end{aligned}$$

In analogy, linearization of *q* is obtained as:

17 $$\begin{aligned} \dfrac{\partial q}{\partial \Delta \boldsymbol{\varepsilon }} &= \dfrac{3}{2} \dfrac{1}{q} \left( \textbf{s}: \dfrac{\partial \textbf{s}}{\partial \Delta \boldsymbol{\varepsilon }} \right) \\&= \dfrac{3}{2} \dfrac{1}{q} \left( \textbf{s}: {\mathbb {D}} + \left( \textbf{s}: \textbf{D} \right) \dfrac{\partial \Delta \phi }{\partial \Delta \boldsymbol{\varepsilon }} \right) . \end{aligned}$$

For the MCC model, linearization of the yield function (1) leads to:

18 $$\begin{aligned} &\dfrac{2q}{M^2} \dfrac{\partial q}{\partial \Delta \boldsymbol{\varepsilon }} + \dfrac{\partial p}{\partial \Delta \boldsymbol{\varepsilon }} \left( p - p_c \right) \\&+ p \left( \dfrac{\partial p}{\partial \Delta \boldsymbol{\varepsilon }} - \dfrac{\partial p_c}{\partial \Delta \boldsymbol{\varepsilon }} \right) = 0. \end{aligned}$$

Equation 18 allows to incorporate (16) and (17) to (18), which then effectively leads to an equation to determine $\dfrac{\partial \Delta \phi }{\partial \Delta \boldsymbol{\varepsilon }}$ for the MCC model:

19 $$\begin{aligned} &\left[ \dfrac{3}{M^2} \textbf{s}: \textbf{D} + \left( 2p - p_c \right) a - p b \right] \dfrac{\partial \Delta \phi }{\partial \Delta \boldsymbol{\varepsilon }} \\&= -\left( 2p - p_c \right) \textbf{A} + p \textbf{B} - \dfrac{3}{M^2} \textbf{s}: {\mathbb {D}}. \end{aligned}$$

For the CASM model, linearization of the yield function (6) leads to:

20 $$\begin{aligned} &\dfrac{1}{p} \left[ \dfrac{1}{\ln R} - N \left( \dfrac{q}{Mp} \right) ^N \right] \dfrac{\partial p}{\partial \Delta \boldsymbol{\varepsilon }} \\&+ \dfrac{N q^{N-1}}{(M p)^N} \dfrac{\partial q}{\partial \Delta \boldsymbol{\varepsilon }} \\&- \dfrac{1}{\ln R} \dfrac{1}{p_c} \dfrac{\partial p_c}{\partial \Delta \boldsymbol{\varepsilon }} = 0, \end{aligned}$$

Substituting (16) and (17) into (20) leads to an equation to determine $\dfrac{\partial \Delta \phi }{\partial \Delta \boldsymbol{\varepsilon }}$ for the CASM model:

21 $$\begin{aligned} \begin{aligned}&\left( \dfrac{a}{p} \left[ \dfrac{1}{\ln R} - N \left( \dfrac{q}{Mp} \right) ^N \right] + \dfrac{3}{2} \dfrac{N q^{N-2} (\textbf{s}: \textbf{D})}{(M p)^N} - \dfrac{1}{\ln R} \dfrac{b}{p_c} \right)\\& \dfrac{\partial \Delta \phi }{\partial \Delta \boldsymbol{\varepsilon }} = - \dfrac{\textbf{A}}{p} \left[ \dfrac{1}{\ln R} + N \left( \dfrac{q}{Mp} \right) ^N \right] \\&+ \dfrac{3}{2} \dfrac{N q^{N-2} (\textbf{s}: {\mathbb {D}})}{(M p)^N} + \dfrac{1}{\ln R} \dfrac{\textbf{B}}{p_c}. \end{aligned} \end{aligned}$$

Solving (19) and (21), respectively, the consistent tangent operator for both models can be calculated using (16) and (14). Details of the derivation of the terms $\textbf{A}, a, \textbf{B}, b, \textbf{D}$ and ${\mathbb {D}}$, are contained in the Appendix  B for the MCC model, and in the Appendix  E for the CASM model.

### Sub-stepping integration scheme

#### Stress integration algorithm

In the sub-stepping integration scheme, the strain increment $\Delta \boldsymbol{\varepsilon }$ is divided into sub-increments

22 $$\begin{aligned} \Delta \boldsymbol{\varepsilon } = \sum _{k=1}^m {}^{k} \Delta \boldsymbol{\varepsilon } \,, \qquad {}^{k} \Delta \boldsymbol{\varepsilon } = \alpha _k \Delta \boldsymbol{\varepsilon } \,, \qquad \sum _{k=1}^m \alpha _k = 1. \end{aligned}$$

where *m* is the number of sub-steps. The stress integration in one sub-step is the same as in the one-step scheme in Sect. 2.3, with the initial state of sub-step $k+1$ defined as the state of the previous sub-step *k*. The stress at the end of the sub-step *k* is denoted as ${^k} \boldsymbol{\sigma }$. The final stress is the stress obtained in sub-step $k=m$: $\boldsymbol{\sigma }_{n+1} = {^m} \boldsymbol{\sigma }_{n+1}$. Evidently, the one-step algorithm is a particular case of the sub-stepping algorithm, with the number of sub-steps set to one.

#### Consistent tangent operator

The tangent operator $\dfrac{ \partial \, {^k} \boldsymbol{\sigma }}{\partial \, {^k} \Delta \boldsymbol{\varepsilon }}$ can be computed using the approach of one-step scheme. Nevertheless, the tangent operator $\dfrac{\partial \boldsymbol{\sigma }}{\partial \Delta \boldsymbol{\varepsilon }}=\dfrac{\partial \, {^m} \boldsymbol{\sigma }}{\partial \Delta \boldsymbol{\varepsilon }}$, which is the total derivative of the final stress state at the end of the load step with respect to the full strain increment, cannot be trivially computed as the sum of the tangent operators in the individual sub-step, since ${^{k+1}} \boldsymbol{\sigma }$ depends on ${^k} \boldsymbol{\sigma }$ and ${^{k+1}} \Delta \boldsymbol{\varepsilon }$. Instead, $\dfrac{\partial \, {^k} \boldsymbol{\sigma }}{\partial \Delta \boldsymbol{\varepsilon }}$ shall be determined for all sub-steps.

From (14), one obtains $\dfrac{\partial \, {^k} \boldsymbol{\sigma }}{\partial \Delta \boldsymbol{\varepsilon }} = \dfrac{\partial \, {^k} \textbf{s}}{\partial \Delta \boldsymbol{\varepsilon }} + \textbf{I} \otimes \dfrac{\partial \, {^k} p}{\partial \Delta \boldsymbol{\varepsilon }}$. $\dfrac{\partial \, {^k} \textbf{s}}{\partial \Delta \boldsymbol{\varepsilon }}$ and $\dfrac{\partial \, {^k} p}{\partial \Delta \boldsymbol{\varepsilon }}$ can be determined recursively, as presented in the following: Firstly, $\dfrac{\partial \, {}^k \textbf{s}}{\partial \, {}^k \Delta \boldsymbol{\varepsilon }}$, $\dfrac{\partial \, {}^k p}{\partial \, {}^k \Delta \boldsymbol{\varepsilon }}$, $\dfrac{\partial \, {}^k p_c}{\partial \, {}^k \Delta \boldsymbol{\varepsilon }}$ with $k=1$ can be determined using the one-step scheme in Sect. 2.3. Applying (22) leads to the consistent linearization of the stresses in sub-step $k=1$: $\dfrac{\partial \, {}^1 \textbf{s}}{\partial \Delta \boldsymbol{\varepsilon }} = \alpha _1 \dfrac{\partial \, {}^1 \textbf{s}}{\partial \, {}^1 \Delta \boldsymbol{\varepsilon }}$, $\dfrac{\partial \, {}^1 p}{\partial \Delta \boldsymbol{\varepsilon }} = \alpha _1 \dfrac{\partial \, {}^1 p}{\partial \, {}^1 \Delta \boldsymbol{\varepsilon }}$ and $\dfrac{\partial \, {}^1 p_c}{\partial \Delta \boldsymbol{\varepsilon }} = \alpha _1 \dfrac{\partial \, {}^1 p_c}{\partial \, {}^1 \Delta \boldsymbol{\varepsilon }}$. Secondly, assuming $\dfrac{\partial \, {}^k \textbf{s}}{\partial \Delta \boldsymbol{\varepsilon }}$, $\dfrac{\partial \, {}^k p}{\partial \Delta \boldsymbol{\varepsilon }}$ and $\dfrac{\partial \, {}^k p_c}{\partial \Delta \boldsymbol{\varepsilon }}$ are already known from the previous sub-step, the linearization in the current sub-step $k+1$ is formulated depending, if the current sub-step is an elastic or an elasto-plastic step:

*Elastic state:*

If the sub-step $(k+1)$ is elastic, the following relations hold:

23 $$\begin{aligned} &{}^{k+1} p = {}^{k} p \, \text {exp} \left( \dfrac{1+e}{\kappa } {}^{k+1} \Delta \epsilon _v \right) \,,\\& {}^{k+1} \textbf{s} = {}^k \textbf{s} + 2 \bar{\mu } \, {}^{k+1} \Delta \boldsymbol{\varepsilon }_d \,,\\& \bar{\mu } = \dfrac{{}^{k+1} p - {}^k p}{{}^{k+1} \Delta \epsilon _v} r. \end{aligned}$$

Taking the linearization of (23) leads to

24 $$\begin{aligned} \dfrac{\partial \, {}^{k+1} p}{\partial \Delta \boldsymbol{\varepsilon }} &= \dfrac{{}^{k+1} p}{{}^{k} p} \dfrac{\partial {}^k p}{\partial \Delta \boldsymbol{\varepsilon }} + \alpha _{k+1} \, {}^{k+1} K \textbf{I} \,,\\ \dfrac{\partial \, {}^{k+1} \textbf{s}}{\partial \Delta \boldsymbol{\varepsilon }} &= \dfrac{\partial \, {}^{k} \textbf{s}}{\partial \Delta \boldsymbol{\varepsilon }} + 2 \alpha _{k+1} \bar{\mu } {\mathbb {I}}_d \\&\quad+ 2 \, {}^{k+1} \Delta \boldsymbol{\varepsilon }_d \otimes \dfrac{\partial \bar{\mu }}{\partial \Delta \boldsymbol{\varepsilon }}, \end{aligned}$$

with

25 $$\begin{aligned} \dfrac{\partial \bar{\mu }}{\partial \Delta \boldsymbol{\varepsilon }} &= \dfrac{\bar{\mu }}{{}^k p} \dfrac{\partial \, {}^k p}{\partial \Delta \boldsymbol{\varepsilon }} \\&\quad+ \alpha _{k+1} \dfrac{{}^{k+1} K r - \bar{\mu }}{{}^{k+1} \Delta \epsilon _v} \textbf{I} \,,\\ {}^{k+1} K &= \dfrac{1+e}{\kappa } {}^{k+1} p. \end{aligned}$$

*Elasto-plastic state:* The linearizations $\dfrac{\partial \, {}^{k+1} \textbf{s}}{\partial \Delta \boldsymbol{\varepsilon }}$, $\dfrac{\partial \, {}^{k+1} p}{\partial \Delta \boldsymbol{\varepsilon }}$ and $\dfrac{\partial \, {}^{k+1} p_c}{\partial \Delta \boldsymbol{\varepsilon }}$ in the plastic state are re-written in terms of $\dfrac{\partial \, {}^{k+1} \Delta \phi }{\partial \Delta \boldsymbol{\varepsilon }}$. To simplify the notation, $\dfrac{\partial \, {}^{k+1} \Delta \phi }{\partial \Delta \boldsymbol{\varepsilon }}$ will be denoted as $\dfrac{\partial \Delta \phi }{\partial \Delta \boldsymbol{\varepsilon }}$ for brevity:

26 $$\begin{aligned} \dfrac{\partial \, {}^{k+1} p}{\partial \Delta \boldsymbol{\varepsilon }} &= \textbf{A} + a \dfrac{\partial \Delta \phi }{\partial \Delta \boldsymbol{\varepsilon }} \,,\\ \dfrac{\partial \, {}^{k+1} p_c}{\partial \Delta \boldsymbol{\varepsilon }} &= \textbf{B} + b \dfrac{\partial \Delta \phi }{\partial \Delta \boldsymbol{\varepsilon }} \,,\\ \dfrac{\partial \, {}^{k+1} \textbf{s}}{\partial \Delta \boldsymbol{\varepsilon }} &= {\mathbb {D}} + \textbf{D} \otimes \dfrac{\Delta \phi }{\Delta \boldsymbol{\varepsilon }}. \end{aligned}$$

The procedure to compute $\dfrac{\partial \Delta \phi }{\partial \Delta \boldsymbol{\varepsilon }}$ is analogous to the one-step scheme for the elasto-plastic state (see Sect. 2.3.2). The derivation of the terms $\textbf{A}, a, \textbf{B}, b, \textbf{D}$ and ${\mathbb {D}}$ for the sub-step $k+1$ can be found in Appendix  C for the MCC model, and Appendix  F for the CASM model. It is noted that, the total derivatives in Eqs. (24) and (26) are updated recursively and not needed to be saved for each sub-steps.

#### Automatic sub-stepping strategy

When the plastic loads enters the softening domain, i.e., on the left side of the critical state line in the *p*-*q* plane, the sub-step increment $\alpha _k$ may need to be adjusted accordingly. In a restart automatic strategy, one can start with a uniform sub-division of the full increment then start over the sub-stepping with a smaller increment if a sub-step fails. This approach may deem costly since it neglects successful sub-steps, and also lead to many sub-steps if the increment needs to be reduced further in the softening regime. Therefore, a smart automatic strategy is devised based on stack approach to adjust the increment as needed and avoid to restart the whole procedure. This strategy is described in Algorithm 1. In contrast to the explicit sub-stepping algorithm presented by Sloan and coworkers [30], which uses explicit equation to integrate the stress and plastic strain, the automatic sub-stepping integration is fully implicit. As a result, each sub-step inherently satisfies the yield criteria and plastic flow rule upon completion, eliminating the need for an error-controlled measure. By comparison, in the explicit sub-stepping algorithm the stress state and plastic consistency are not enforced at the end of a sub-step. Consequently, the sub-step size must be adjusted continuously through an error-controlled procedure to ensure accuracy and stability.

**Algorithm 1:**
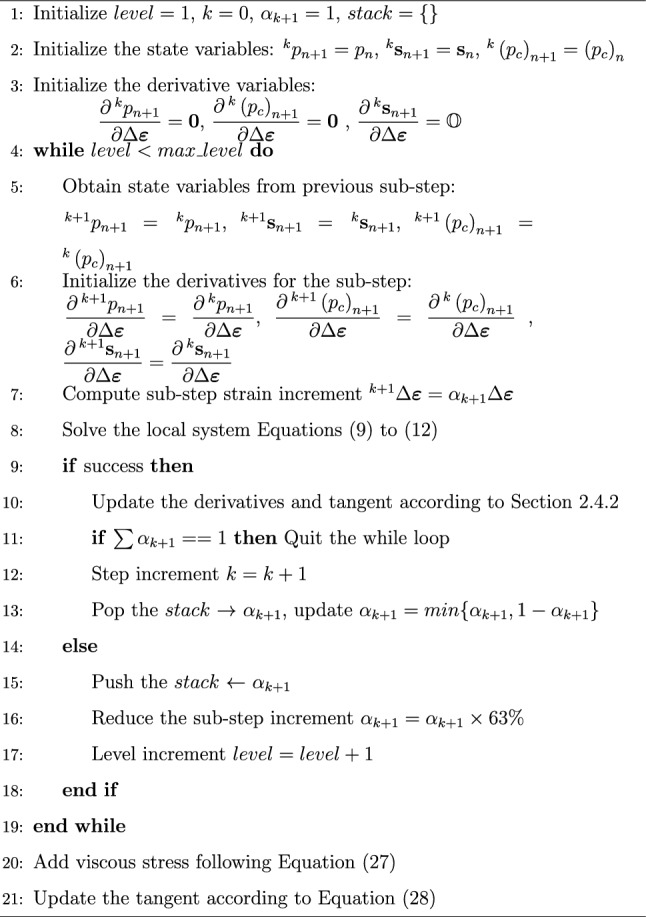
Automatic sub-stepping strategy

The automatic sub-stepping strategy takes advantage of a stack [9] to store the last non-converged sub-step increment, and revert back to use that increment when the last sub-step converges. Furthermore, a reduction ratio of $37 \%$ is applied when the sub-step does not converge. This ratio is heuristic in order not to reduce the sub-step drastically. After each converged sub-step, the new increment is computed based on current distance to 1, i.e., full step, not to exceed the full strain increment. A maximum number of reduction level can be prescribed to avoid excessive sub-stepping. In that case, the structure likely enters the failure mode locally and the simulation cannot be continued. In the next global iteration, the successful sub-stepping profile corresponding to the previous iteration is repeated to maintain the continuity of the consistent tangent.

#### Viscous damping stabilization

Although the consistent linearization of the sub-stepping strategy preserves the quadratic convergence, it may not ensure the global convergence, especially when many integration points enter the softening domain, see e.g., Fig. 9. In this case, the global tangent becomes ill-posed and loses its positive definiteness. To improve on that, a slight pertubation can be added to the stress to improve the stability of the global iteration procedure. This is achieved by adding a viscous component to the stress, such as

27 $$\begin{aligned} \boldsymbol{\sigma }^{pert} = \boldsymbol{\sigma } + \gamma \dot{\boldsymbol{\varepsilon }} \end{aligned}$$

in which $\gamma$ is the viscous damping factor, and is usually small $(10^{-5}< \gamma < 10^{-3})$ to not affect the accuracy of the analysis. The consistent linearization of the viscous damping component is expressed as

28 $$\begin{aligned} {\mathbb {C}}^{pert} = {\mathbb {C}} + \gamma {\mathbb {I}}_s \end{aligned}$$

where ${\mathbb {I}}_s$ is the fourth-order symmetric tensor [17].

## Numerical examples

### Triaxial test of a soil sample

**Fig. 1:**
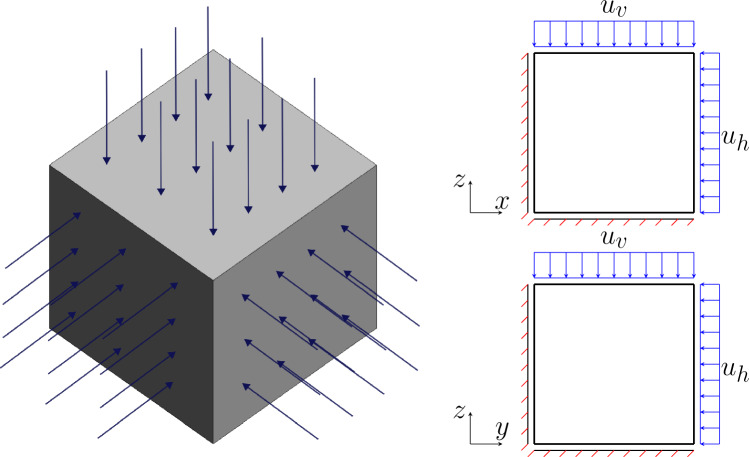
Triaxial test example: illustration of geometry and boundary conditions

In the first example, the drained compressive triaxial test on a cube sample is performed to validate the accuracy of the implicit sub-stepping algorithm. The geometry and boundary condition can be seen in Fig. 1, in which the top surface is applied with prescribed displacement $u_v$ and the sides are applied with prescribed displacement $u_h$. The geometry is meshed with single hexahedra element with 8 nodes. The test is performed on 3 clay samples with the initial stress state as normally consolidated (case 1, $\text {OCR}=1$), lightly over-consolidated (case 2, $\text {OCR}=2$) and highly over-consolidated (case 3, $\text {OCR}=5$). The proportional loading scheme is selected as the controlled stress path, for which the analytical solution exists. For MCC model, the analytical solution subjected to drained proportional loading can be found in [20], and for the CASM model, in Appendix  G. In the proportional loading scheme, the load is controlled in such a way that $\dot{q} = k \dot{p}$ (*k*: loading rate). To produce the stress path that matches with the analytical solution, the axial strain $\epsilon _a$ is controlled to match with the displacement history of the analytical solution. It is noted that $\epsilon _a=\dfrac{1}{3} \epsilon _v + \epsilon _q$, in which $\epsilon _v$ is the volumetric strain and $\epsilon _q$ is the shear strain.

In the simulation, the following parameters are used for the MCC model: { $\lambda =0.066$, $\kappa =0.0077$, $M=1.2$, $e=1.788$, $\nu =0.3$ }; and for the CASM model: { $\lambda =0.066$, $\kappa =0.0077$, $N=3$, $M=1.2$, $R=2.0$, $e=1.788$, $\nu =0.3$ }. The implicit sub-stepping algorithm is enforced to run with 2 sub-steps to verify the correctness of the implementation. The loading rate is selected as $k=3$.

#### Results with MCC model

**Fig. 2:**
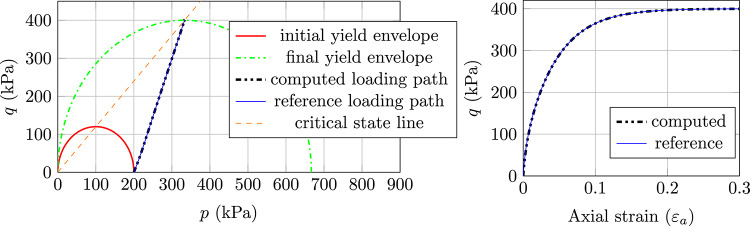
Triaxial test example, MCC model, case 1: Left) computed stress path and Right) Load–displacement curve

**Fig. 3:**
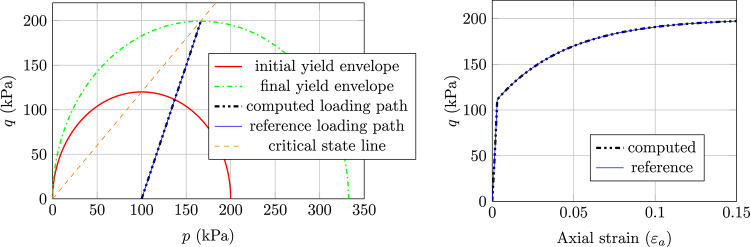
Triaxial test example, MCC model, case 2: Left) computed stress path and Right) Load–displacement curve

**Fig. 4:**
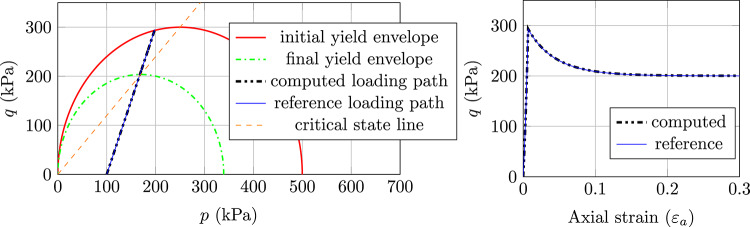
Triaxial test example, MCC model, case 3: Left) computed stress path and Right) Load–displacement curve

The results, as presented in Fig. 2 for normally consolidated soil, Fig. 3 for lightly consolidated soil and Fig. 4 for highly consolidated soil show excellent agreement with the analytical results. In case 1, the stress state starts from the initial yield envelope and stops at the consolidation line as expected. The shear stress asymptotically converges and the soil sample cannot sustain further load. Figure 2 shows the computed stress path (Left) and the according load–displacement curve (Right). The same behaviour is observed for case 2, where the stress state starts from the elastic domain and enters the plastic state after hitting the initial yield envelope (see Fig. 3 (Left)). From that point, the soil sample starts to harden and the shear stress asymptotically converges (see Fig. 3 (Right)). Case 1 and case 2 show that the implicit sub-stepping algorithm works well on the dry side of the modified Cam-Clay model.

In case 3, the softening behaviour on the wet side of the MCC is tested. As can be seen in Fig. 4 (Left), the stress state starts in the elastic domain and after hitting the initial yield envelope, it starts to soften and the shear stress asymptotically converges (see Fig. 4 (Right)). The stress state ultimately stops at the consolidation line as expected.

#### Results with CASM model

**Fig. 5:**
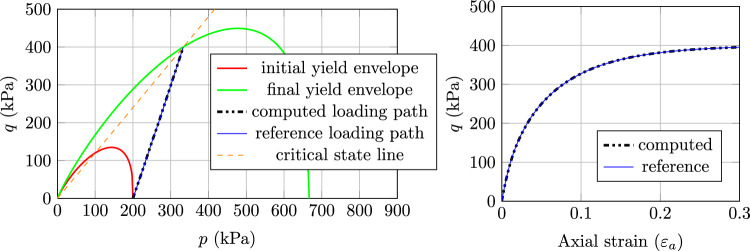
Triaxial test example, CASM model, case 1: Left) computed stress path and Right) Load–displacement curve

**Fig. 6:**
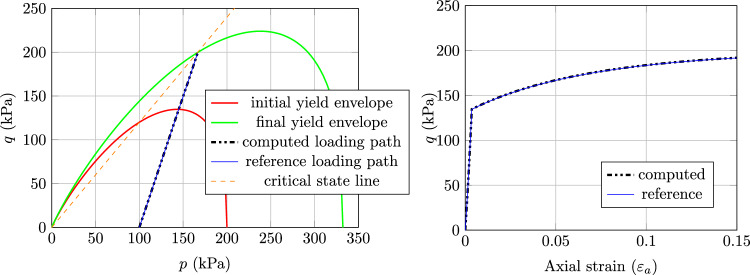
Triaxial test example, CASM model, case 2: Left) computed stress path and Right) Load–displacement curve

**Fig. 7:**
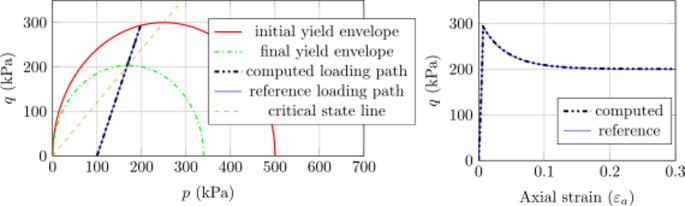
Triaxial test example, CASM model, case 3: Left) computed stress path and Right) Load–displacement curve

The implicit sub-stepping algorithm also work expectedly with the CASM model in the triaxial test, as shown in Fig. 5 (case 1), Fig. 6 (case 2) and Fig. 7 (case 3). For each case, the evolution of the stress state is analogous to the respective case of MCC model. Nevertheless, the stress state hits the initial yield envelope early for case 3 and lately in case 2 due to the shape of the yield surface. Similar to the MCC case, the implicit sub-stepping algorithm also performs well on the wet side of the CASM model.

### Excavation of a soil block

**Fig. 8:**
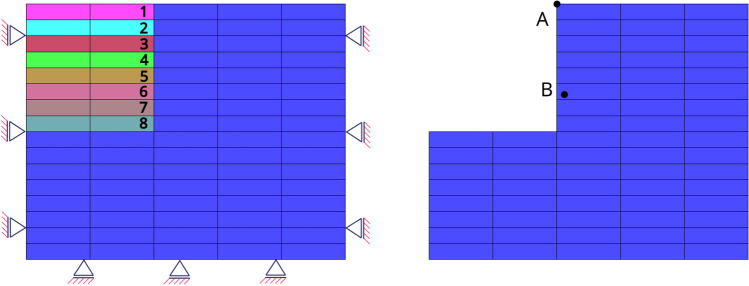
Block excavation example: illustration of geometry, computational mesh, and boundary condition. The numbered layers on the top-left corner represent the excavation layers

In this example, the simulation of the excavation process of a soil block is presented to demonstrate the use of the implicit sub-stepping integration algorithm of MCC and CASM model in a practical application. The geometry (rectangular block of $5 \times 4$ m) and material parameters for this example are taken from [3]. The computational mesh and boundary conditions of the block are illustrated in Fig. 8, where the original and final excavating state are shown. The mesh uses quadrilateral 9-node discretization, amounts to a total number of 80 elements. For MCC model, the material parameters read: $\lambda =0.37$, $\kappa =0.054$, $M=1.4$, $e=2.52$ and $\nu =0.35$. For CASM model, additional parameters are set, in which $N=1.4$ and $R=2.0$, to approximately match the corresponding MCC model. In difference to the original example, the over-consolidation ratio ($\text {OCR}$) is preset to 1.7 for the half upper part and $\text {OCR}=1.0$ for the half lower part of the soil block. The initial stress of the soil are generated by $K_0$-procedure with $K_0=1.2$ and the soil density is $\rho =1732 \, kg/m^3$. The preconsolidation pressure are generated from the initial stress using the yield criteria and $\text {OCR}$ as following:

- for MCC:
  29 $$\begin{aligned} p_c = \text {OCR} \left( p + \dfrac{q^2}{M^2 p} \right) . \end{aligned}$$
- for CASM:
  30 $$\begin{aligned} p_c = \text {OCR} \, p \, \exp \left[ \ln R \left( \dfrac{q}{M p} \right) ^N \right] . \end{aligned}$$

Using the same analysis sequence as the original example [3], the analysis with different number of excavation steps are simulated. With a total of 8 element layers representing the excavation domain, the number of excavation steps are simulated as 1, 2, 4 and 8 lifts corresponding to 8, 4, 2 and 1 number of excavated layers per step, respectively, see Fig. 8 (Left). The excavation sequence for the simulation with different number of lifts can be found in Table 1. The simulation with MCC converges with only one step, nevertheless, the sub-stepping has to be turned on for the CASM simulation to overcome the convergence difficulty. The displacements of the monitoring point A, see Fig. 8 (Right), corresponding to different lifts, together with the number of average iterations in an excavation step, are reported in Table 2. It is noted that, the displacements are presented in *mm* and the tolerance for convergence is $10^{-11}$. From Table 2, the displacement results slightly differs between MCC and CASM for the same type of lift procedure. This is expected since the yield surface are approximately matched and the plastic flow functions are different.

**Table 1:** Excavation of a soil block: excavation sequence for different number of lifts

No. of lifts	Lift 1	Lift 2	Lift 3	Lift 4	Lift 5	Lift 6	Lift 7	Lift 8
1	{1,2,3,4,5,6,7,8}							
2	{1,2,3,4}	{5,6,7,8}						
4	{1,2}	{3,4}	{5,6}	{7,8}				
8	{1}	{2}	{3}	{4}	{5}	{6}	{7}	{8}

**Table 2:** Excavation of a soil block: displacements at the reference point A and the average number of iterations per step for different number of lifts

No. of lifts	MCC			CASM		
	$d_x$	$d_y$	Avg. iter/lift	$d_x$	$d_y$	Avg. iter/lift
1	−29.05	−16.15	8	−30.04	−15.22	16
2	−28.55	−16.27	8	−29.48	−15.25	8.5
4	−28.03	−16.15	7.2	−28.8	−14.92	11.2
8	−27.84	−16.02	6.4	−28.6	−14.79	6.6

The global iteration of CASM simulation takes significantly more steps than the MCC counterpart. Nevertheless, the global convergence is not attainable without sub-stepping. Table 3 shows the sub-stepping details of simulation for one number of lift, i.e., all layers are excavated at once, in which the element number, the integration point number within the element, the sub-step increments and the failed trial sub-steps according to Algorithm 1 are recorded. It can be observed in Table 3 that, there are not so many points requiring sub-stepping and the number of failed sub-steps is not significant. Hence, the additional computatinal cost of sub-stepping is negligible, yet, its application was necessary for robust and reliable solution.

**Table 3:** Excavation of a soil block: sub-stepping information for each iteration in each lift in simulation using CASM for case No. of lifts = 1

It	(Element no., point no.)	Sub-step increments	No. of failed sub-steps
2	(43, 3)	{ 0.63, 0.37 }	1
3	(16, 1)	{ 0.63, 0.37 }	1
4	(16, 5)	{ 0.63, 0.37 }	1
	(38, 2)	{ 0.63, 0.37 }	1
5	(16, 5)	{ 0.63, 0.37 }	1
6	(16, 1)	{ 0.63, 0.146853, 0.140583, 0.0825644 }	4
7	(16, 1)	{ 0.63, 0.146853, 0.140583, 0.0825644 }	4
	(38, 2)	{ 0.3969, 0.379953, 0.223147 }	3
	(43, 2)	{ 0.63, 0.37 }	1
8	(16, 2)	{ 0.63, 0.37 }	1
	(16, 4)	{ 0.63, 0.37 }	1
9	(16, 8)	{ 0.63, 0.37 }	1
10	(16, 1)	{ 0.3969, 0.150803, 0.150803, 0.23937, 0.0621229 }	6
11	(16, 1)	{ 0.63, 0.37 }	1

**Fig. 9:**
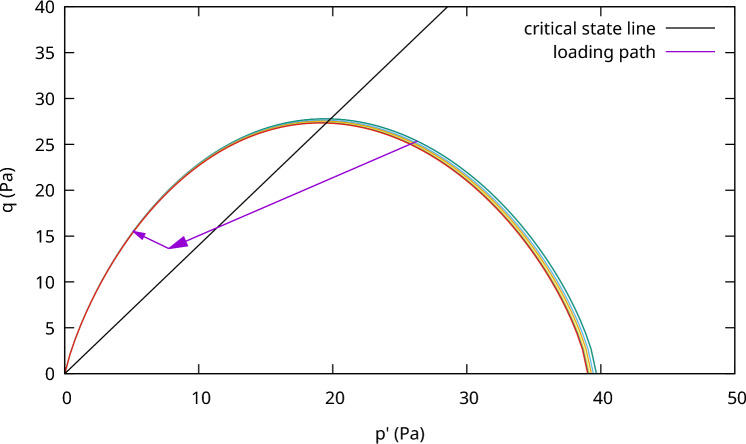
Excavation of a soil block: loading path at point B when softening with four lift simulation

Table 4 reports the convergence of the residual for each excavation step, denoted as lift, in each analysis with different number of total lifts. It can be seen that, the convergence rate is at linear at the beginning, however once it enters the convergence radius, the rate becomes quadratic again. This validates the correctness of the consistent linearization scheme of the sub-stepping strategy. For the case of four lift simulation, the global number of iteration is 24, which is due to the local softening at point B(x = 2.11, y = 2.53), denoted in Fig. 8 (Right), and shown in Fig. 9 for the local stress path. Due to the loss of ellipticity, additional viscous damping of $10^{-5}$ is activated to stabilize the convergence. The increasing number of global iteration in this case should not be seen as a drawback of the sub-stepping algorithm, but quite the contrary, as if the global solution strategy would have been applied, it would be required to reduce the load step or applying stress relaxation procedure to overcome the softening region. This equivalently leads to significantly more global iterations in the last lift.

**Table 4:** Excavation of a soil block: convergence of the simulation using CASM according to different number of lifts

	No. of lifts = 1	No. of lifts = 2		No. of lifts = 4				No. of lifts = 8							
It	Lift 1	Lift 1	Lift 2	Lift 1	Lift 2	Lift 3	Lift 4	Lift 1	Lift 2	Lift 3	Lift 4	Lift 5	Lift 6	Lift 7	Lift 8
0	3.53E+01	1.75E+01	2.10E+01	8.75E+00	9.61E+00	1.12E+01	1.32E+01	4.37E+00	4.56E+00	4.95E+00	5.53E+00	6.33E+00	7.26E+00	8.26E+00	9.28E+00
1	1.08E+01	5.59E+00	7.46E+00	2.98E+00	3.39E+00	4.35E+00	7.71E+00	1.57E+00	1.58E+00	1.75E+00	1.87E+00	2.51E+00	4.49E+00	6.11E+00	7.91E+00
2	3.86E+00	1.99E+00	2.63E+00	8.25E−01	1.23E+00	1.33E+00	9.13E+00	2.69E−01	5.03E−01	7.87E−01	5.75E−01	6.23E−01	1.51E+00	2.96E+00	4.94E+00
3	1.40E+00	5.47E−01	9.76E−01	1.54E−01	2.83E−01	2.15E−01	7.27E+00	1.74E−02	7.21E−02	7.62E−02	6.92E−02	6.22E−02	6.44E−01	2.40E+00	5.44E+00
4	2.52E+00	7.50E−02	1.83E−01	7.05E−03	1.83E−02	1.41E−02	1.02E+01	1.13E−04	1.73E−03	1.54E−03	1.14E−03	3.17E−04	3.73E−02	2.45E−01	1.61E+00
5	6.63E−01	3.29E−03	5.22E−02	2.37E−05	9.38E−05	5.45E−05	6.18E+00	7.07E−09	1.18E−06	7.92E−07	3.42E−07	2.16E−08	4.23E−04	3.44E−03	1.05E−01
6	2.33E−01	1.47E−05	8.15E−05	5.07E−10	3.61E−09	9.65E−10	9.61E+00	8.74E−13	7.48E−13	1.73E−12	2.03E−12	2.16E−12	3.08E−08	6.61E−07	4.97E−04
7	6.88E−02	4.40E−10	1.97E−07	8.93E−14	1.07E−13	1.69E−13	6.47E+00						8.97E−12	1.16E−11	1.57E−08
8	9.09E−01	1.05E−13	2.30E−11				9.45E+00							1.46E−13	2.92E−12
9	1.50E−01		3.23E−13				6.60E+00								
10	7.06E−02						9.31E+00								
11	2.39E−01						6.89E+00								
12	2.46E−02						1.01E+01								
13	1.08E−03						6.70E+00								
14	1.83E−06						7.72E+00								
15	2.03E−10						8.09E+00								
16	1.26E−13						5.95E+00								
17							5.23E+00								
18							2.62E+00								
19							1.19E+00								
20							4.23E−02								
21							5.38E−03								
22							2.15E−06								
23							1.84E−10								
24							3.25E−13								

### Slope stability analysis

**Fig. 10:**
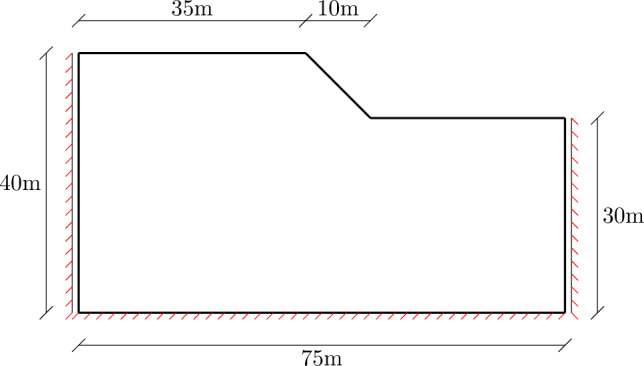
Slope stability example: illustration of geometry and boundary conditions

**Fig. 11:**
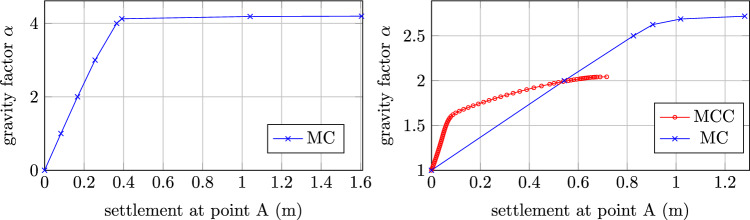
Load displacement curve at sampling point of slope stability using (Left) the parameters as in [18] and (Right) the calibrated parameters following MCC

The slope is a typical geotechnical structure which can develop localized deformation under gravity load. This makes the slope vulnerable to failure under extreme condition, such as when the pore water pressure builds up. Therefore, it is crucial to evaluate the existing shear strength in comparison with the reduced strength at failure. In this study, the safety factor of a slope is estimated using the MCC model, integrated by the sub-stepping procedure presented in Sect. 2. The geometry and material parameters are based on the benchmark example from [18]. The dimension of the slope and boundary condition are visualized in Fig. 10. In essence, the simulation sequence is performed by gradually increasing the gravity until the slope fails, which interprets into the numerical computation by non-convergence of the material subroutine. The final load factor is then considerred as the safety factor of the slope. For the sake of comparison, the simulation using perfect Mohr-Coulomb (MC), with parameters $E=20$ MPa, $\nu =0.49$, $\phi =\psi =20^\circ$ and $c=50$ kPa is performed. The slope fails at the gravity factor $\alpha _{lim}=4.195$, which is closed to the results reported in [18]. The result is shown as load–displacement curve at the sampling point A, located at the top of the slope, in Fig. 11 (Left).

Since the stiffness in MCC is pressure dependent, it is not possible to directly compare the two models in terms of stability analysis. Therefore, a reverse approach is proposed. We first compute the limited load factor for MCC, using the parameters as in Sect. 3.1. We then select the average pressure from the top left region of the slope, which is representative for the unfailed region of the slope, to estimate the MC parameters as

31 $$\begin{aligned} \phi &= \text {asin} \left( \dfrac{3M}{6 + M} \right) \,,\\ E &= 3 \dfrac{1+e}{\kappa } (1-2\nu ) p \,,\\ c &= q - p \tan {\phi } \end{aligned}$$

**Fig. 12:**
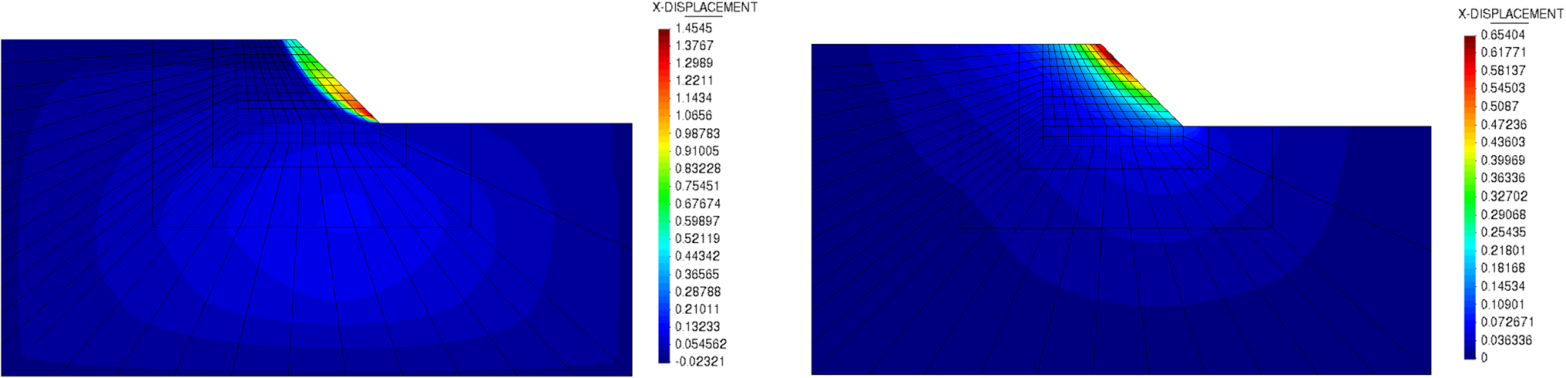
Slope stability example: displacement in x-direction for Mohr-Coulomb (Left) and MCC with substepping (Right)

The attained safety factor, given the pressure for the limited load that triggers yielding with MC is $\sim 2.72$ and with MCC is $\sim 2.044$, as shown in Fig. 11 (Right). One also can see the horizontal displacement of the slope at the limited load in Fig. 12. Providing that the stiffness is non-homogeneous in MCC and a nonlinear constitutive relation is used in the elastic region, the difference in the results between MC and MCC is justifiable. In addition, it can be seen that both MC and MCC model can predict the localized strain region. Although the localized region is not as clear with MCC as with the MC analysis, it can be improved by large strain analysis, which is currently out of the scope of this paper. Furthermore, automatic sub-stepping is triggered during MCC analysis in the top right region (marked with red displacement in Fig. 12 (Right)). The analyis continues until the structure loses its bearing capacity, marked by horizontal slope of the load–displacement curve, see Fig. 11 (Right).

In the MCC analysis, the initial incremental gravity factor is set as one and further divided in half when the global convergence is not attained. This reveals the global stepping pattern described in Table 5, which constitutes a global load factor of 2.044. Table 5 also shows that, simulation without sub-stepping triggers smaller step sizes at the beginning and eventually requires much smaller step size plus significant amount of global steps to converge to the same load factor. In terms of CPU time, the simulation without sub-stepping takes $\sim 2.64s$, meanwhile the simulation with sub-stepping using the same global stepping takes $\sim 1.54s$. The analysis is performed using 1 thread on a computer with CPU Intel Core i9-13950HX and 64GB of memory.

**Table 5:** Slope stability analysis: global stepping pattern for analysis with (WS) and without (WO) local sub-stepping

	$2^0$	$2^{-1}$	$2^{-2}$	$2^{-3}$	$2^{-4}$	$2^{-5}$	$2^{-6}$	$2^{-7}$	$2^{-8}$	$2^{-9}$	$2^{-10}$	$2^{-11}$	$2^{-12}$	$2^{-13}$	$2^{-14}$	$2^{-15}$	Sum
WS	1	1		1		9	7	2	1	3	3						2.044
WO	1		2		2		25		2	1	2	1		2	1	1	2.028

### Tunnel excavation example

#### NATM tunnel in 2D

In the next example, a simulation of tunnel excavation employing New Austrian Tunnelling Method (NATM) is presented. The objective of this example is to illustrate the advantage of implicit sub-stepping algorithm over the one-step algorithm in which the latter one fails, which means the implicit sub-stepping algorithm does not converge in one step. The criteria of the local failure is either the non-convergence of the local Newton–Raphson iteration to solve the local nonlinear system, or the inadmissibility of the resulting stress state, i.e. $p \le 0 \vee q < 0 \vee p_c \le 0$. NATM is an tunnelling approach that takes into account the load bearing of surrounding soil by gradually removing the material so that the soil strength could be mobilized. At the end of the excavation, shotcrete is typically used to seal off the open face and to stabilize the ground state. For more details of the NATM, the readers are referred to [11, 13].

**Fig. 13:**
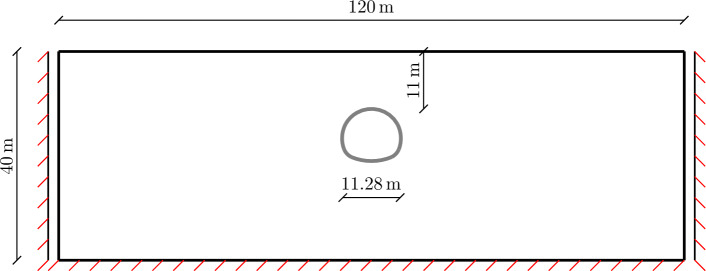
Tunnel excavation example: illustration of geometry and boundary conditions

Figure 13 shows the geometry and boundary conditions of the problem at hand. They are adopted from the tutorial 7 of ADONIS geotechnical software [15]. The following parameters are adopted for the MCC and CASM model: $\lambda =0.147$, $\kappa =0.06$, $M=1.05$, $e=3.56$ and $\nu =0.3$. For CASM, additional parameters are used: $N=1.4$ and $R=2.0$ to approximate the respective MCC model. The geometry of the problem is meshed by 5231 quadratic quadrilateral element (9-node quad) and the full integration scheme with 9 integration points per element is used.

Because the number of required sub-steps is not known in advance, a restart automatic stepping scheme is used in accordance with the implicit sub-stepping algorithm. If the integration at a sub-step fails, the number of sub-steps at the integration point will be doubled and the sub-stepping integration is started over. Tolerance of $10^{-8}$ is adopted for the local iteration and tolerance of $10^{-10}$ is adopted for the global Newton–Raphson loop.

Analogous to the previous example, the initial stress state is generated using $K_0$-procedure with $K_0=0.5$, $\rho =1732 \, kg/m^3$ and the initial preconsolidation pressure profile is computed using Eq. (29) for MCC and Eq. (30) for CASM respectively. The over-consolidation ratio is set to $\text {OCR}=2.0$.

To simulate the excavation process, a stress relaxation approach is adopted. At first, the elements in the excavation domain are deactivated and the corresponding traction is applied on the tunnel wall to maintain the equilibrium. The traction is computed from the initial stress field within the soil. In the second step, the traction is reduced to 40 %. Subsequently, the shotcrete, represented by beam elements is installed in the current configuration. The parameters for the shotcrete beam element are $E=1.56e6 \, kPa$, $\nu =0.2$ and the thickness is $t=0.1 \, m$. Finally, after the shotcrete is installed, the applied traction on tunnel wall is removed. The plane strain condition is assumed in the analysis.

**Fig. 14:**
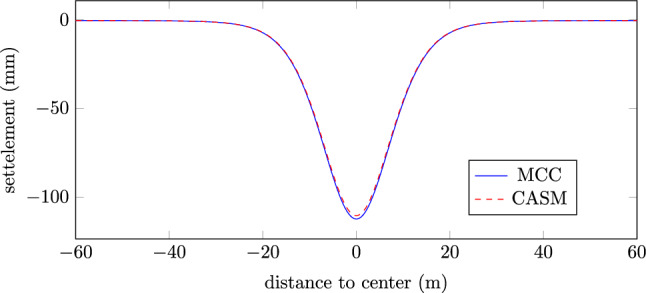
2D NATM excavation example: settlement profile on top surface

The settlement profile on the top surface, after the final step, is shown in Fig. 14. As one shall expect, the results of MCC and CASM is very closed, due to the yield surfaces are matched.

In terms of convergence, the classical one-step algorithm fails for both MCC and CASM in the loading step when the traction is reduced. In the implicit sub-stepping algorithm, a total of 70 sub-steps for MCC and 392 sub-steps for CASM is required at those failed plastic points, recorded in the one-step analysis. The statistics of the sub-stepping algorithm are summarized in Table 6. In the table, PP denotes the plastic point, and SPP is the plastic point where the sub-stepping is required. The average number of sub-steps is computed by summing all number of sub-steps at SPP and divide by the number of SPPs.

**Table 6:** Excavation of a soil block: displacements at the reference point A and the convergence of the implicit sub-stepping integration algorithm for different number of lifts

Traction	No. of PP	No. of SPP	No. of sub-steps		
			Min	Avg	Max
*MCC*					
70%	45,450	0	0	0	0
40%	45,450	13	2	5.4	32
26.67%	45,450	0	0	0	0
13.33%	45,450	0	0	0	0
0%	45,450	0	0	0	0
*CASM*					
70%	45,450	0	0	0	0
40%	45,450	87	2	4.5	16
26.67%	45,450	0	0	0	0
13.33%	45,450	0	0	0	0
0%	45,450	0	0	0	0

In this example, the sub-stepping integration is required only in the step where the reduced traction occurs. Although the number of SPP is not high, it is crucial for the continuation of the analysis where one-step algorithm fails and cannot continue.

#### TBM excavation in 3D

**Fig. 15:**
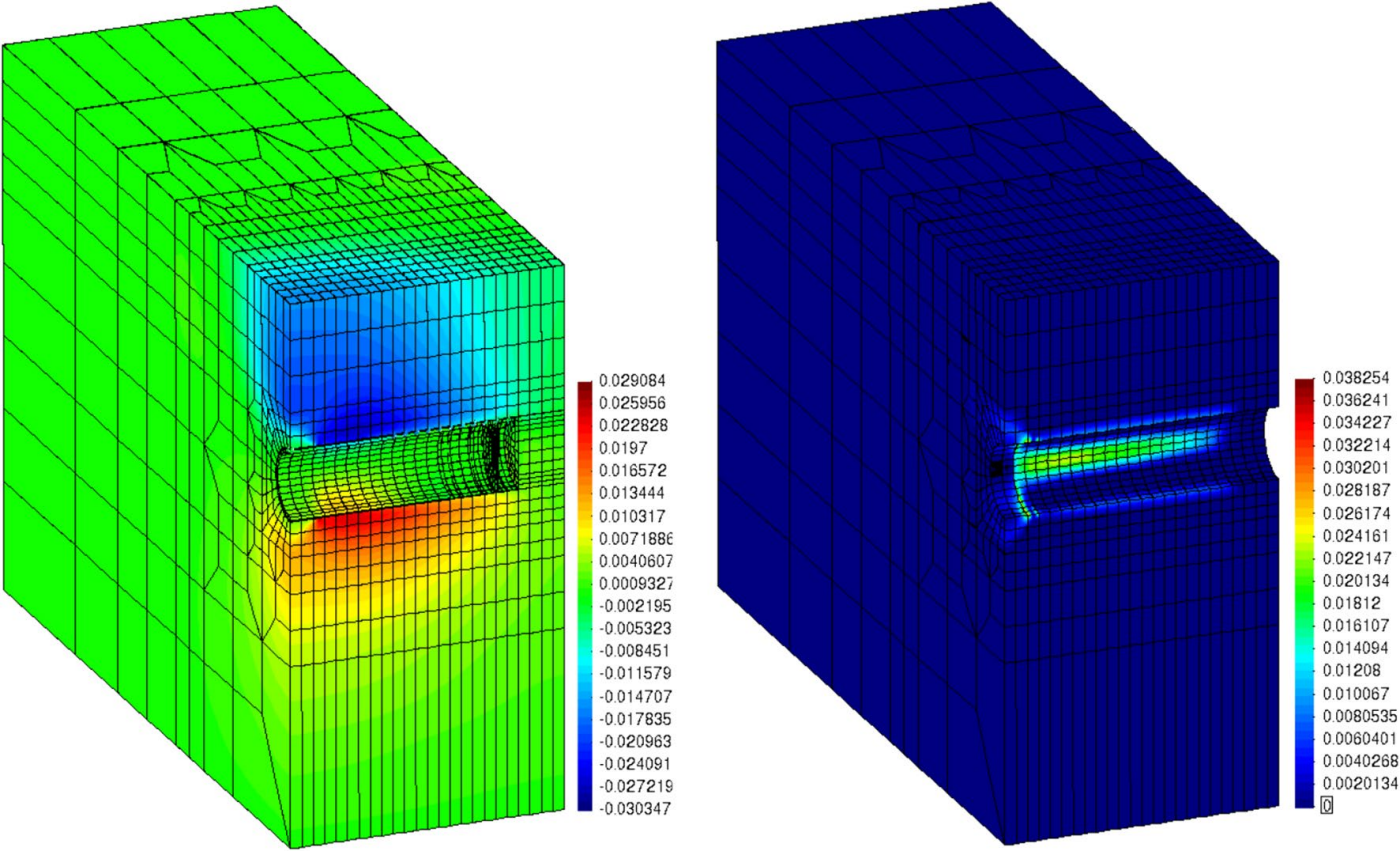
3D TBM excavation example: z-displacement (Left) and accumulated plastic strain (Right) at time = 6.29 h

**Fig. 16:**
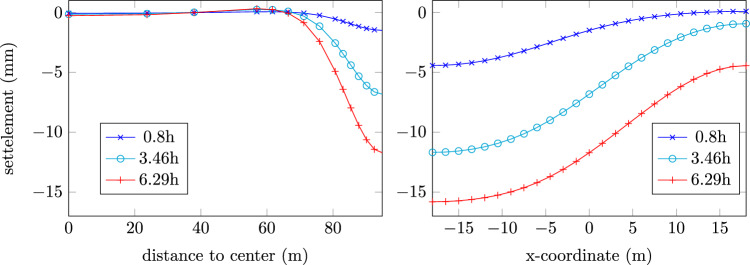
3D TBM excavation example: transaxial settlement (Left) and axial settlement (Right) in different time steps

In the last and final example, a 3D excavation example with Tunnel Boring Machine (TBM) is simulated with CASM model. The drained condition is implied and second-order small strain finite element is used for the ground. For the details of the computational model for mechanized tunnelling used in this example, the reader is referred to [8, 16]. The following parameters are adopted for the CASM model: { $\lambda =0.2$, $\kappa =0.02$, $N=3$, $M=1.2$, $R=2.0$, $e=4.0$, $\nu =0.3$ }. Furthermore, $K_0=1.2$ is assumed for insitu stress calculation and overconsolidation ratio $OCR=1.05$ is used to initialize the preconsolidation pressure field for critical state model. The augmented Lagrangian contact algorithm [14] is used to properly account for the interaction between the TBM and the ground. The displacement field of the last step is visualized in Fig. 15 (Left) and settlement profile, corresponding with start (time=0.8h), middle (time=3.46h) and end (time=6.29h) of the excavation sequence, is plotted in Fig. 16. A maximum settlement of $\sim 12$ mm is observed in the middle section. The accumulated plastic strain localizes around the tunnel side, crown and invert, as can be seen in Fig. 15 (Right). This is due to the stress redistribution when the ground removal is developed. Furthermore, the typical uplift effect, when the tunnel invert contracts and the crown settles into the tunnel chamber, is also observed. This demonstrates that the implemented critical state model effectively captures the qualitative aspects of soil-structure interaction in TBM excavation.

## Conclusions

This work presents an implicit sub-stepping algorithm that greatly improves the computability of the critical state soil models. The sub-stepping stress integration is not new, nevertheless this is the first time a sub-stepping algorithm is fully linearized to be used with the implicit Newton–Raphson iteration for the critical state models, particularly for the popular Modified Cam-Clay and Clay-And-Sand models. The validation examples show that the sub-stepping integration algorithm can reproduce the analytical solution and the results exhibit quadratic convergence behavior. Importantly, the implicit sub-stepping algorithm is able to integrate the plastic points where the traditional one-step scheme fails. This greatly enhances the robustness of the critical state models in geotechnical analysis. Since the plastic potential function in the formulation is used independently with the yield function, the consistent linearization fully supports non-asscociative plasticity. The proposed integration scheme can be extended to other class of soil models, such as bounding Cam-Clay, Barcelona-Basic model, etc. This will be the subject for future development.

## Data Availability

No datasets were generated or analysed during the current study.

## Appendix

### A. Derivation of the local nonlinear system of the critical state model

From Eq. (5), one can derive the incremental plastic strains as

32 $$\begin{aligned} \Delta \varepsilon ^p_v = \theta \dfrac{\partial g}{\partial p} \,, \quad \Delta \boldsymbol{\varepsilon }^p_d = \theta \dfrac{\partial g}{\partial q} \hat{\textbf{n}} \end{aligned}$$

From Eq. (2), we can deduce

33 $$\begin{aligned} p_{n+1}&= p_n \exp \left[ \dfrac{1+e}{\kappa } \Delta \varepsilon ^e_v \right] \end{aligned}$$

34 $$\begin{aligned} \textbf{s}_{n+1}&= \textbf{s}_{n} + 2 \bar{\mu } \Delta \boldsymbol{\varepsilon }^e_d \end{aligned}$$

in which $\bar{\mu }$ is the average of the shear modulus over the load step, which reads

35 $$\begin{aligned} \bar{\mu } = \bar{K} r \,, \quad \bar{K} = \dfrac{p_{n+1} - p_n}{\Delta \varepsilon ^e_v} \end{aligned}$$

It is noted that $\Delta \boldsymbol{\varepsilon }^e_d = \Delta \boldsymbol{\varepsilon }_d - \Delta \boldsymbol{\varepsilon }^p_d$, hence

36 $$\begin{aligned} \textbf{s}_{n+1} = \textbf{s}_{n} + 2 \bar{\mu } \Delta \boldsymbol{\varepsilon }_d - 2 \bar{\mu } \theta \dfrac{\partial g}{\partial q} \hat{\textbf{n}} \end{aligned}$$

From Eq. (36), one can compute $\hat{\textbf{n}}$ from the trial stress as

37 $$\begin{aligned} \hat{\textbf{n}} = \dfrac{\textbf{s}_{n} + 2 \bar{\mu } \Delta \boldsymbol{\varepsilon }_d}{\Vert \textbf{s}_{n} + 2 \bar{\mu } \Delta \boldsymbol{\varepsilon }_d \Vert } \end{aligned}$$

Ultimately, it allows to compute the new stress $\boldsymbol{\sigma }_{n+1}$ directly by $p_{n+1}$ and $q_{n+1}$ such as

38 $$\begin{aligned} \boldsymbol{\sigma }_{n+1} = p_{n+1} \textbf{I} + \sqrt{\dfrac{2}{3}} q_{n+1} \hat{\textbf{n}} \end{aligned}$$

The scalar version of Eq. (34) provides the equation to compute $q_{n+1}$, which is $h_2$ in Eq. (10). Analogously, Eq. (33) leads to $h_1$ in Eq. (9).

By taking the integration of Eq. (4) over the load step, one obtains $h_3$ in Eq. (11). Finally, $h_4$, i.e., Eq. (12), enforces the yield condition during plastic loading.

### B. Linearization of the one-step scheme, modified Cam-Clay model

Taking the linearization of (9) and (11), one obtains:

39 $$\begin{aligned} \dfrac{\partial p}{\partial \Delta \boldsymbol{\varepsilon }}&= \dfrac{1+e}{\kappa } p \dfrac{\partial \Delta \varepsilon ^e_v}{\partial \Delta \boldsymbol{\varepsilon }} , \end{aligned}$$

40 $$\begin{aligned} \dfrac{\partial p_c}{\partial \Delta \boldsymbol{\varepsilon }}&= \theta p_c \left[ \Delta \phi \left( 2\dfrac{\partial p}{\partial \Delta \boldsymbol{\varepsilon }} - \dfrac{\partial p_c}{\partial \Delta \boldsymbol{\varepsilon }} \right) + \left( 2p - p_c \right) \dfrac{\partial \Delta \phi }{\partial \Delta \boldsymbol{\varepsilon }} \right] . \end{aligned}$$

Equation 40 is equivalent to:

41 $$\begin{aligned} \dfrac{\partial p_c}{\partial \Delta \boldsymbol{\varepsilon }} = \dfrac{\theta p_c}{1 + \theta p_c \Delta \phi } \left[ 2\Delta \phi \dfrac{\partial p}{\partial \Delta \boldsymbol{\varepsilon }} + \left( 2p - p_c \right) \dfrac{\partial \Delta \phi }{\partial \Delta \boldsymbol{\varepsilon }} \right] . \end{aligned}$$

It is noted that $\Delta \varepsilon ^p_v = \Delta \phi (2p-p_c)$ and $\Delta \varepsilon ^e_v = \Delta \epsilon _v - \Delta \phi (2p-p_c)$, hence:

42 $$\begin{aligned} \dfrac{\partial \Delta \varepsilon ^e_v}{\partial \Delta \boldsymbol{\varepsilon }} &= \textbf{I} - \Delta \phi \left( 2 \dfrac{\partial p}{\partial \Delta \boldsymbol{\varepsilon }} - \dfrac{\partial p_c}{\partial \Delta \boldsymbol{\varepsilon }} \right) \\&\quad- \left( 2p - p_c \right) \dfrac{\partial \Delta \phi }{\partial \Delta \boldsymbol{\varepsilon }}. \end{aligned}$$

Substituting (42) and (41) into (39) leads to:

43 $$\begin{aligned} &\left( 1 + \dfrac{1+e}{\kappa } p \dfrac{2 \Delta \phi }{1 + \theta p_c \Delta \phi } \right) \dfrac{\partial p}{\partial \Delta \boldsymbol{\varepsilon }} \\&= \dfrac{1+e}{\kappa } p \left[ \textbf{I} - \dfrac{2p - p_c}{1 + \theta p_c \Delta \phi } \dfrac{\partial \Delta \phi }{\partial \Delta \boldsymbol{\varepsilon }} \right] . \end{aligned}$$

Equation (43) can be rewritten as:

44 $$\begin{aligned} \begin{aligned}&\dfrac{\partial p}{\partial \Delta \boldsymbol{\varepsilon }} = \textbf{A}_1 + a_1 \dfrac{\partial \Delta \phi }{\partial \Delta \boldsymbol{\varepsilon }}, \\& K = \dfrac{1+e}{\kappa } p, \quad a_2 = 1 + \left( \theta p_c + 2K \right) \Delta \phi , \\&\textbf{A} = \textbf{A}_1 = \dfrac{1 + \theta p_c \Delta \phi }{a_2} K \textbf{I}, \\& a = a_1 = -\dfrac{2p - p_c}{a_2} K. \end{aligned} \end{aligned}$$

Replaces (44) into (41), one obtains:

45 $$\begin{aligned} &\dfrac{\partial p_c}{\partial \Delta \boldsymbol{\varepsilon }} = \textbf{B}_1 + b_1 \dfrac{\partial \Delta \phi }{\partial \Delta \boldsymbol{\varepsilon }}, \\& \textbf{B} = \textbf{B}_1 = \dfrac{2\theta p_c \Delta \phi }{a_2} K \textbf{I}, \\& b = b_1 = \dfrac{2p-p_c}{a_2} \theta p_c. \end{aligned}$$

The deviatoric pressure reads:

46 $$\begin{aligned} \textbf{s} &= \eta \left( \textbf{s}_n + 2\bar{\mu } \Delta \boldsymbol{\varepsilon }_d \right) \,, \\ \bar{\mu } &= \dfrac{p - p_n}{\Delta \epsilon _v^e} r \,, \\ \eta &= \left( 1 + 6\bar{\mu }\dfrac{\Delta \phi }{M^2} \right) ^{-1}. \end{aligned}$$

Taking the linearization of (46) with respect to $\Delta \boldsymbol{\varepsilon }$, one obtains:

47 $$\begin{aligned} &\dfrac{\partial \textbf{s}}{\partial \Delta \boldsymbol{\varepsilon }} = \left( \textbf{s}_n + 2\bar{\mu }\Delta \boldsymbol{\varepsilon }_d \right) \otimes \dfrac{\partial \eta }{\partial \Delta \boldsymbol{\varepsilon }} \\&+ 2\eta \Delta \boldsymbol{\varepsilon }_d \otimes \dfrac{\partial \bar{\mu }}{\partial \Delta \boldsymbol{\varepsilon }} \\&+ 2\eta \bar{\mu } \dfrac{\partial \Delta \boldsymbol{\varepsilon }_d}{\partial \Delta \boldsymbol{\varepsilon }}. \end{aligned}$$

In (47), this relation holds: $\dfrac{\partial \Delta \boldsymbol{\varepsilon }_d}{\partial \Delta \boldsymbol{\varepsilon }} = {\mathbb {I}}_d = {\mathbb {I}} - \dfrac{1}{3} \textbf{I} \otimes \textbf{I}$, where ${\mathbb {I}}$ is the fourth-order identity tensor [17]. The linearization $\dfrac{\partial \bar{\mu }}{\partial \Delta \boldsymbol{\varepsilon }}$ and $\dfrac{\partial \eta }{\partial \Delta \boldsymbol{\varepsilon }}$ come as following:

48 $$\begin{aligned} \dfrac{\partial \bar{\mu }}{\partial \Delta \boldsymbol{\varepsilon }} &= \textbf{D}_1 + d_1 \dfrac{\partial \Delta \phi }{\partial \Delta \boldsymbol{\varepsilon }} \,, \\ \textbf{D}_1 &= \dfrac{K r - \bar{\mu }}{K \Delta \epsilon _v^e} \textbf{A}_1 \,, \\ d_1 &= \dfrac{K r - \bar{\mu }}{K \Delta \epsilon _v^e} a_1, \end{aligned}$$

49 $$\begin{aligned} \dfrac{\partial \eta }{\partial \Delta \boldsymbol{\varepsilon }} &= \textbf{D}_2 + d_2 \dfrac{\partial \Delta \phi }{\partial \Delta \boldsymbol{\varepsilon }} \,, \\ \textbf{D}_2 &= -6 \eta ^2 \dfrac{\Delta \phi }{M^2} \textbf{D}_1 \,, \\ d_2 &= -\dfrac{6 \eta ^2}{M^2} \left( \Delta \phi d_1 + \bar{\mu } \right) . \end{aligned}$$

Substituting (48) and (49) into (47) results in $\dfrac{\partial \textbf{s}}{\partial \Delta \boldsymbol{\varepsilon }}$:

50 $$\begin{aligned} \dfrac{\partial \textbf{s}}{\partial \Delta \boldsymbol{\varepsilon }} = {\mathbb {D}} + \textbf{D}_3 \otimes \dfrac{\partial \Delta \phi }{\partial \Delta \boldsymbol{\varepsilon }}, \end{aligned}$$

In which

51 $$\begin{aligned} {\mathbb {D}}&= \textbf{s}_n \otimes \textbf{D}_2 + \Delta \boldsymbol{\varepsilon }_d \otimes 2\left( \bar{\mu } \textbf{D}_2 + \eta \textbf{D}_1 \right) + 2\eta \bar{\mu } {\mathbb {I}}_d , \nonumber \\ \textbf{D}&= \textbf{D}_3 = d_2 \textbf{s}_n + 2\left( \bar{\mu } d_2 + \eta d_1 \right) \Delta \boldsymbol{\varepsilon }_d . \end{aligned}$$

### C. Linearization of the sub-stepping scheme, modified Cam-Clay model

Taking linearization of (9) and (11) for the sub-step $(k+1)$, with the remark that linearization of ${}^k p$ and ${}^k p_c$ do not vanish, one obtains:

52 $$\begin{aligned} \dfrac{\partial \, {}^{k+1}p}{\partial \Delta \boldsymbol{\varepsilon }} &= \dfrac{{}^{k+1}p}{{}^{k}p} \dfrac{\partial \, {}^{k}p}{\partial \Delta \boldsymbol{\varepsilon }} \\&\quad+ \dfrac{1+e}{\kappa } {}^{k+1} p \dfrac{\partial \, {}^{k+1}\Delta \varepsilon ^e_v}{\partial \Delta \boldsymbol{\varepsilon }}, \end{aligned}$$

and

53 $$\begin{aligned} &\dfrac{\partial \, {}^{k+1} p_c}{\partial \Delta \boldsymbol{\varepsilon }} = \dfrac{{}^{k+1} p_c}{{}^{k} p_c} \dfrac{\partial \, {}^{k} p_c}{\partial \Delta \boldsymbol{\varepsilon }} \\&+ \theta \, {}^{k+1} p_c \left[ \Delta \phi \left( 2\dfrac{\partial \, {}^{k+1} p}{\partial \Delta \boldsymbol{\varepsilon }} - \dfrac{\partial \, {}^{k+1} p_c}{\partial \Delta \boldsymbol{\varepsilon }} \right) + \left( 2 \, {}^{k+1} p - {}^{k+1} p_c \right) \dfrac{\partial \Delta \phi }{\partial \Delta \boldsymbol{\varepsilon }} \right] . \end{aligned}$$

It is noted that ${}^{k+1} \Delta \varepsilon ^e_v = {}^{k+1} \Delta \epsilon _v - \Delta \phi (2 \, {}^{k+1} p - {}^{k+1} p_c)$, hence:

54 $$\begin{aligned} \dfrac{\partial \, {}^{k+1} \Delta \varepsilon ^e_v}{\partial \Delta \boldsymbol{\varepsilon }} &= \alpha _{k+1} \textbf{I} - \Delta \phi \left( 2 \dfrac{\partial \, {}^{k+1} p}{\partial \Delta \boldsymbol{\varepsilon }} - \dfrac{\partial \, {}^{k+1} p_c}{\partial \Delta \boldsymbol{\varepsilon }} \right) \\&\quad- \left( 2 \, {}^{k+1} p - {}^{k+1} p_c \right) \dfrac{\partial \Delta \phi }{\partial \Delta \boldsymbol{\varepsilon }}. \end{aligned}$$

Using (54) and solve (52) and (53) with respect to variable $\dfrac{\partial \, {}^{k+1}p}{\partial \Delta \boldsymbol{\varepsilon }}$ and $\dfrac{\partial \, {}^{k+1}p_c}{\partial \Delta \boldsymbol{\varepsilon }}$ leads to:

55 $$\begin{aligned} \dfrac{\partial \, {}^{k+1}p}{\partial \Delta \boldsymbol{\varepsilon }} &= \textbf{A}_1 + a_1 \dfrac{\partial \Delta \phi }{\partial \Delta \boldsymbol{\varepsilon }} \,, \\ \dfrac{\partial \, {}^{k+1}p_c}{\partial \Delta \boldsymbol{\varepsilon }} &= \textbf{B}_1 + b_1 \dfrac{\partial \Delta \phi }{\partial \Delta \boldsymbol{\varepsilon }}. \end{aligned}$$

In which

56 $$\begin{aligned} \textbf{A}&= \textbf{A}_1 = \dfrac{1 + \theta \, {}^{k+1} p_c \Delta \phi }{a_2} \left( \dfrac{{}^{k+1} p}{{}^k p} \dfrac{\partial \, {}^{k} p}{\partial \Delta \boldsymbol{\varepsilon }} + \alpha _{k+1} {}^{k+1}K \textbf{I} \right) \\&\quad+ \dfrac{{}^{k+1} K \Delta \phi }{a_2} \dfrac{{}^{k+1} p_c}{{}^k p_c} \dfrac{\partial \, {}^{k} p_c}{\Delta \boldsymbol{\varepsilon }} , \nonumber \\ a&= a_1 = - \dfrac{2 \, {}^{k+1} p - {}^{k+1} p_c}{a_2} {}^{k+1} K , \nonumber \\ a_2&= 1 + \left( \theta \, {}^{k+1} p_c + 2 \, {}^{k+1} K \right) \Delta \phi . \end{aligned}$$

57 $$\begin{aligned} \textbf{B}&= \textbf{B}_1 = \dfrac{2 \theta \, {}^{k+1} p_c \Delta \phi }{a_2} \left( \dfrac{{}^{k+1} p}{{}^k p} \dfrac{\partial \, {}^{k} p}{\partial \Delta \boldsymbol{\varepsilon }} + \alpha _{k+1} {}^{k+1}K \textbf{I} \right) \\&\quad+ \dfrac{1 + 2\, {}^{k+1} K \Delta \phi }{a_2} \dfrac{{}^{k+1} p_c}{{}^k p_c} \dfrac{\partial \, {}^{k} p_c}{\Delta \boldsymbol{\varepsilon }} , \end{aligned}$$

58 $$\begin{aligned} b&= b_1 = \dfrac{\theta \, {}^{k+1} p_c}{a_2} \left( 2 \, {}^{k+1} p - {}^{k+1} p_c \right) , \end{aligned}$$

59 $$\begin{aligned} {}^{k+1} K&= \dfrac{1 + e}{\kappa } {}^{k+1} p . \end{aligned}$$

From Eq. (52):

60 $$\begin{aligned} \begin{aligned}\dfrac{\partial \, {}^{k+1}\Delta \varepsilon ^e_v}{\partial \Delta \boldsymbol{\varepsilon }} &= \dfrac{1}{{}^{k+1} K} \left( \dfrac{\partial \, {}^{k+1} p}{\partial \Delta \boldsymbol{\varepsilon }} - \dfrac{{}^{k+1} p}{{}^{k} p} \dfrac{\partial \, {}^{k} p}{\partial \Delta \boldsymbol{\varepsilon }} \right) \\&= \textbf{C}_1 + c_1 \dfrac{\partial \Delta \phi }{\partial \Delta \boldsymbol{\varepsilon }} \,, \\\textbf{C}_1 &= \dfrac{1}{{}^{k+1}K} \left( \textbf{A}_1 - \dfrac{{}^{k+1} p}{{}^{k} p} \dfrac{\partial \, {}^{k} p}{\partial \Delta \boldsymbol{\varepsilon }} \right) \,, \\ c_1 &= \dfrac{a_1}{{}^{k+1}K} \end{aligned} \end{aligned}$$

The deviatoric pressure reads:

61 $$\begin{aligned} {}^{k+1} \textbf{s} &= \eta \left( {}^k \textbf{s} + 2 \bar{\mu } \, {}^{k+1} \Delta \boldsymbol{\varepsilon }_d \right) \,, \\ \bar{\mu } &= \dfrac{{}^{k+1} p - {}^k p}{{}^{k+1} \, \Delta \epsilon _v^e} r \,, \\ \eta &= \left( 1 + 6\bar{\mu }\dfrac{\Delta \phi }{M^2} \right) ^{-1}. \end{aligned}$$

It is therefore:

62 $$\begin{aligned} \dfrac{\partial \bar{\mu }}{\partial \Delta \boldsymbol{\varepsilon }} &= \dfrac{r}{{}^{k+1} \, \Delta \epsilon _v^e} \left( \dfrac{\partial \, {}^{k+1}p}{\partial \Delta \boldsymbol{\varepsilon }} - \dfrac{\partial \, {}^{k}p}{\partial \Delta \boldsymbol{\varepsilon }} \right) \\&\quad- \dfrac{\bar{\mu }}{{}^{k+1} \Delta \epsilon _v^e} \dfrac{\partial \, {}^{k+1}\Delta \varepsilon ^e_v}{\partial \Delta \boldsymbol{\varepsilon }}. \end{aligned}$$

Replace (52) and (60) into (62), one obtains:

63 $$\begin{aligned} \begin{aligned}\dfrac{\partial \bar{\mu }}{\partial \Delta \boldsymbol{\varepsilon }} &= \textbf{D}_1 + d_1 \dfrac{\partial \Delta \phi }{\partial \Delta \boldsymbol{\varepsilon }} \,, \\ \textbf{D}_1 &= \dfrac{r \textbf{A}_1 - \bar{\mu }\textbf{C}_1}{{}^{k+1} \Delta \varepsilon ^e_v} \\&\quad- \dfrac{r}{{}^{k+1} \Delta \varepsilon ^e_v} \dfrac{\partial \, {}^{k} p}{\partial \Delta \boldsymbol{\varepsilon }} \,, \\d_1 &= \dfrac{r a_1 - \bar{\mu }c_1}{{}^{k+1}\Delta \varepsilon ^e_v}. \end{aligned} \end{aligned}$$

The derivation of $\dfrac{\partial \eta }{\partial \Delta \boldsymbol{\varepsilon }}$ is similar to (49), which reads:

64 $$\begin{aligned} \begin{aligned}\dfrac{\partial \eta }{\partial \Delta \boldsymbol{\varepsilon }} &= -\dfrac{6 \eta ^2}{M^2} \left( \Delta \phi \dfrac{\partial \bar{\mu }}{\partial \Delta \boldsymbol{\varepsilon }} + \bar{\mu } \dfrac{\partial \Delta \phi }{\partial \Delta \boldsymbol{\varepsilon }} \right) \\&= \textbf{D}_2 + d_2 \dfrac{\partial \Delta \phi }{\partial \Delta \boldsymbol{\varepsilon }} \,, \\\textbf{D}_2 &= -\dfrac{6 \eta ^2 \Delta \phi }{M^2} \textbf{D}_1 \,, \\ d_2 &= -\dfrac{6\eta ^2}{M^2} \left( \Delta \phi d_1 + \bar{\mu } \right) . \end{aligned} \end{aligned}$$

The linearization of deviatoric pressure ${}^{k+1} \textbf{s}$ follows by:

65 $$\begin{aligned} \begin{aligned} \dfrac{\partial \, {}^{k+1} \textbf{s}}{\partial \Delta \boldsymbol{\varepsilon }}&= \left( {}^k \textbf{s} + 2 \bar{\mu } \, {}^{k+1} \Delta \boldsymbol{\varepsilon }_d \right) \otimes \dfrac{\partial \eta }{\partial \Delta \boldsymbol{\varepsilon }} \\&\quad+ \eta \dfrac{\partial \, {}^k \textbf{s}}{\partial \Delta \boldsymbol{\varepsilon }} \\&\quad+ 2 \eta \, {}^{k+1} \Delta \boldsymbol{\varepsilon }_d \otimes \dfrac{\partial \bar{\mu }}{\partial \Delta \boldsymbol{\varepsilon }} \\&\quad+ 2 \eta \bar{\mu } \dfrac{\partial \, {}^{k+1} \Delta \boldsymbol{\varepsilon }_d}{\partial \Delta \boldsymbol{\varepsilon }} \\&= {\mathbb {D}} + \textbf{D}_3 \otimes \dfrac{\Delta \phi }{\Delta \boldsymbol{\varepsilon }}, \end{aligned} \end{aligned}$$

In which

66 $$\begin{aligned} {\mathbb {D}}&= \left( {}^k\textbf{s} + 2 \bar{\mu } \, {}^{k+1} \Delta \boldsymbol{\varepsilon }_d \right) \otimes \textbf{D}_2 \\&\quad+ \eta \dfrac{\partial \, {}^k \textbf{s}}{\partial \Delta \boldsymbol{\varepsilon }} \\&\quad+ 2 \eta \, {}^{k+1} \Delta \boldsymbol{\varepsilon }_d \otimes \textbf{D}_1 + 2 \alpha _{k+1} \eta \bar{\mu } {\mathbb {I}}_d , \nonumber \\ \textbf{D}&= \textbf{D}_3 = d_2 \left( {}^k\textbf{s} + 2 \bar{\mu } \, {}^{k+1} \Delta \boldsymbol{\varepsilon }_d \right) \\&\quad+ 2 \eta d_1 \, {}^{k+1} \Delta \boldsymbol{\varepsilon }_d . \end{aligned}$$

### D. Solution of the local nonlinear system, CASM model

In order to derive the solution of the local nonlinear system, necessary derivatives of the yield function and plastic potential are given to support for further derivation:

67 $$\begin{aligned} \begin{aligned}f_{p} &= \dfrac{\partial f}{\partial p} = - \dfrac{N q^N}{M^N p^{N+1}} + \dfrac{1}{p \ln R} \,, \\ f_{q} &= \dfrac{\partial f}{\partial q} = \dfrac{N q^{N-1}}{M^N p^N} \,, \\f_{p_c} &= \dfrac{\partial f}{\partial p_c} = - \dfrac{1}{p_c \ln R} \,, \end{aligned} \end{aligned}$$

68 $$\begin{aligned} g_p &= \dfrac{\partial g}{\partial p} = 3 \dfrac{3+2M}{3p + 2q} - 3 \dfrac{3-M}{3p - q} \,, \\ g_q &= \dfrac{\partial g}{\partial q} = 2 \dfrac{3+2M}{3p+2q} + \dfrac{3-M}{3p - q} \,, \end{aligned}$$

69 $$\begin{aligned} \begin{aligned}&g_{pp} = \dfrac{\partial ^2 g}{\partial p^2} = -9 \dfrac{3+2M}{(3p+2q)^2} + 9 \dfrac{3-M}{(3p-q)^2} \,, \\&g_{qq} = \dfrac{\partial ^2 g}{\partial q^2} = -4 \dfrac{3+2M}{(3p+2q)^2} + \dfrac{3-M}{(3p-q)^2} \,, \end{aligned} \end{aligned}$$

70 $$\begin{aligned} g_{pq} = \dfrac{\partial ^2 q}{\partial p \partial q} = -6 \dfrac{3+2M}{(3p+2q)^2} - 3 \dfrac{3-M}{(3p-q)^2}. \end{aligned}$$

Additional terms are defined as:

71 $$\begin{aligned} \begin{aligned}K &= \dfrac{1+e}{\kappa } p \,, \\ \bar{\mu } &= K r \,, \\ \eta &= \left( 1 + 3\bar{\mu } \dfrac{\Delta \phi }{q} g_q \right) ^{-1} \,, \\\hat{\textbf{n}} &= \dfrac{\textbf{s}_n + 2\bar{\mu } \Delta \boldsymbol{\varepsilon }_d}{\Vert \textbf{s}_n + 2\bar{\mu } \Delta \boldsymbol{\varepsilon }_d \Vert }. \end{aligned} \end{aligned}$$

The flow rule reads:

72 $$\begin{aligned} \begin{aligned}\Delta \varepsilon ^p_v &= \Delta \phi \, g_p \,, \\ \Delta \varepsilon ^e_v &= \Delta \epsilon _v - \Delta \varepsilon ^p_v \,, \\\Delta \boldsymbol{\varepsilon }^p_d &= \sqrt{\dfrac{3}{2}} \Delta \phi \, g_q \hat{\textbf{n}} \,, \\ \Delta \boldsymbol{\varepsilon }^e_d &= \Delta \boldsymbol{\varepsilon }_d - \Delta \boldsymbol{\varepsilon }^p_d. \end{aligned} \end{aligned}$$

The derivatives of $\bar{\mu }$ and $\eta$ read:

73 $$\begin{aligned} \begin{aligned}\bar{\mu }_p &= \dfrac{\partial \bar{\mu }}{\partial p} = \dfrac{1}{\Delta \varepsilon ^e_v} \left( r + \bar{\mu } \Delta \phi \, g_{pp} \right) \,, \\ \bar{\mu }_q &= \dfrac{\partial \bar{\mu }}{\partial q} = \dfrac{\bar{\mu }}{\Delta \varepsilon ^e_v} \Delta \phi \, g_{pq} \,, \\\bar{\mu }_{p_c} &= \dfrac{\partial \bar{\mu }}{\partial p_c} = 0 \,, \\ \bar{\mu }_{\Delta \phi } &= \dfrac{\partial \bar{\mu }}{\partial \Delta \phi } = \dfrac{\bar{\mu }}{\Delta \varepsilon ^e_v} g_{p} \,, \end{aligned} \end{aligned}$$

74 $$\begin{aligned} \begin{aligned} \eta _p&= \dfrac{\partial \eta }{\partial p} = - 3 \dfrac{\Delta \phi }{q} \left( \bar{\mu }_p g_q + \bar{\mu } g_{pq} \right) \eta ^2 \,, \\ \eta _q&= \dfrac{\partial \eta }{\partial p} = - 3 \dfrac{\Delta \phi }{q} \left( \left( \bar{\mu }_q - \dfrac{\bar{\mu }}{q} \right) g_q + \bar{\mu } g_{qq} \right) \eta ^2 \,, \\ \bar{\eta }_{p_c}&= \dfrac{\partial \eta }{\partial p_c} = 0 \,, \\ \bar{\eta }_{\Delta \phi } &= \dfrac{\partial \eta }{\partial \Delta \phi } = - \dfrac{3}{q} \left( \bar{\mu } + \bar{\mu }_{\Delta \phi } \Delta \phi \right) g_q \eta ^2. \end{aligned} \end{aligned}$$

The necessary derivatives to solver local system (9) (10) (11) (12) are given as follow:

$$\begin{aligned} \begin{aligned}h_{1,1} &= \dfrac{\partial h_1}{\partial p} = 1 + K \Delta \phi \, g_{pp} \,, \\ h_{1,2} &= \dfrac{\partial h_1}{\partial q} = K \Delta \phi \, g_{pq} \,, \\h_{1,3} &= \dfrac{\partial h_1}{\partial p_c} = 0 \,, \\ h_{1,4} &= \dfrac{\partial h_1}{\partial \Delta \phi } = K g_{p}. \end{aligned} \end{aligned}$$

$$\begin{aligned} \begin{aligned} h_{2,1}&= \dfrac{\partial h_2}{\partial p} = - \sqrt{\dfrac{3}{2}} \eta _p \Vert \textbf{s}_n + 2\bar{\mu } \Delta \boldsymbol{\varepsilon }_d \Vert \\&\quad- 2 \sqrt{\dfrac{3}{2}} \eta \bar{\mu }_p \hat{\textbf{n}}: \Delta \boldsymbol{\varepsilon }_d \,, \\ h_{2,2}&= \dfrac{\partial h_2}{\partial q} = 1 - \sqrt{\dfrac{3}{2}} \eta _q \Vert \textbf{s}_n + 2\bar{\mu } \Delta \boldsymbol{\varepsilon }_d \Vert \\&\quad- 2 \sqrt{\dfrac{3}{2}} \eta \bar{\mu }_q \hat{\textbf{n}}: \Delta \boldsymbol{\varepsilon }_d \,, \\ h_{2,3}&= \dfrac{\partial h_2}{\partial p_c} = - 2 \sqrt{\dfrac{3}{2}} \eta \bar{\mu }_{p_c} \hat{\textbf{n}}: \Delta \boldsymbol{\varepsilon }_d \,, \\ h_{2,4}&= \dfrac{\partial h_2}{\partial \Delta \phi } = - \sqrt{\dfrac{3}{2}} \eta _{\Delta \phi } \Vert \textbf{s}_n + 2\bar{\mu } \Delta \boldsymbol{\varepsilon }_d \Vert \\&\quad- 2 \sqrt{\dfrac{3}{2}} \eta \bar{\mu }_{\Delta \phi } \hat{\textbf{n}}: \Delta \boldsymbol{\varepsilon }_d \,, \end{aligned} \end{aligned}$$

$$\begin{aligned} \begin{aligned}h_{3,1} &= \dfrac{\partial h_3}{\partial p} = - \theta p_c \Delta \phi \, g_{pp} \,, \\ h_{3,2} &= \dfrac{\partial h_3}{\partial q} = - \theta p_c \Delta \phi \, g_{pq} \,, \\h_{3,3} &= \dfrac{\partial h_3}{\partial p_c} = 1 \,, \\ h_{3,4} &= \dfrac{\partial h_3}{\partial \Delta \phi } = - \theta p_c \, g_{p} \,, \end{aligned} \end{aligned}$$

$$\begin{aligned} h_{4,1} &= \dfrac{\partial h_4}{\partial p} = f_p \,, \\ h_{4,2} &= \dfrac{\partial h_4}{\partial q} = f_q \,, \\ h_{4,3} &= \dfrac{\partial h_4}{\partial p_c} = f_{p_c} \,, \\ h_{4,4} &= \dfrac{\partial h_4}{\partial \Delta \phi } = 0. \end{aligned}$$

### E. Linearization of the one-step scheme, CASM model

Taking the linearization of (9), one obtains:

75 $$\begin{aligned} \dfrac{\partial p}{\partial \Delta \boldsymbol{\varepsilon }} &= K \dfrac{\partial \Delta \varepsilon ^e_v}{\partial \Delta \boldsymbol{\varepsilon }} \,, \\ K &= \dfrac{1+e}{\kappa } p. \end{aligned}$$

It is noted that $\Delta \varepsilon ^e_v = \Delta \epsilon _v - \Delta \phi g_p$, hence:

76 $$\begin{aligned} \dfrac{\partial \Delta \varepsilon ^e_v}{\partial \Delta \boldsymbol{\varepsilon }} &= \textbf{I} - \Delta \phi g_{pp} \dfrac{\partial p}{\partial \Delta \boldsymbol{\varepsilon }} \\&\quad- \Delta \phi g_{pq} \dfrac{\partial q}{\partial \Delta \boldsymbol{\varepsilon }} - g_p \dfrac{\partial \Delta \phi }{\partial \Delta \boldsymbol{\varepsilon }}. \end{aligned}$$

From (75) and (76), one can rewrite $\dfrac{\partial p}{\partial \Delta \boldsymbol{\varepsilon }}$ as dependent term of $\dfrac{\partial q}{\partial \Delta \boldsymbol{\varepsilon }}$ and $\dfrac{\partial \Delta \phi }{\partial \Delta \boldsymbol{\varepsilon }}$:

77 $$\begin{aligned} \dfrac{\partial p}{\partial \Delta \boldsymbol{\varepsilon }} &= \textbf{A}_1 + a_1 \dfrac{\partial \Delta \phi }{\partial \Delta \boldsymbol{\varepsilon }} \\&\quad+ a_2 \dfrac{\partial q}{\partial \Delta \boldsymbol{\varepsilon }} \,,\quad \textbf{A}_1 = a_3 K \textbf{I} \,, \\ a_1 &= -a_3 K g_p \,, \quad a_2 = -a_3 K \Delta \phi g_{pq} \,, \\ a_3 &= \left( 1 + K \Delta \phi g_{pp} \right) ^{-1}. \end{aligned}$$

Taking the linearization of (11):

78 $$\begin{aligned} \dfrac{\partial p_c}{\partial \Delta \boldsymbol{\varepsilon }} &= \theta p_c \left( \textbf{I} - \dfrac{\partial \Delta \varepsilon ^e_v}{\partial \Delta \boldsymbol{\varepsilon }} \right) \\&= \textbf{B}_1 + b_1 \dfrac{\partial \Delta \phi }{\partial \Delta \boldsymbol{\varepsilon }} + b_2 \dfrac{\partial q}{\partial \Delta \boldsymbol{\varepsilon }} \,, \\ \textbf{B}_1 &= \dfrac{\theta p_c}{K} \left( K \textbf{I} - \textbf{A}_1 \right) \,, \\ b_1 &= -\dfrac{\theta p_c}{K} a_1 \,, \quad b_2 = -\dfrac{\theta p_c}{K} a_2. \end{aligned}$$

The deviatoric pressure reads:

79 $$\begin{aligned} \textbf{s} &= \eta \left( \textbf{s}_n + 2 \bar{\mu } \Delta \boldsymbol{\varepsilon }_d \right) \,, \\ \bar{\mu } &= \dfrac{p - p_n}{\Delta \epsilon _v^e} r \,, \\ \eta &= \left( 1 + 3 \bar{\mu }\dfrac{\Delta \phi }{q} g_{q} \right) ^{-1}. \end{aligned}$$

The linearization of $\bar{\mu }$ comes as follows:

80 $$\begin{aligned} \dfrac{\partial \bar{\mu }}{\partial \Delta \boldsymbol{\varepsilon }} &= \textbf{D}_1 + d_1 \dfrac{\partial \Delta \phi }{\partial \Delta \boldsymbol{\varepsilon }} \\&\quad+ d_2 \dfrac{\partial q}{\partial \Delta \boldsymbol{\varepsilon }} \,, \\ \textbf{D}_1 &= \dfrac{K r - \bar{\mu }}{K \Delta \epsilon _v^e} \textbf{A}_1 \,,\\ d_1 &= \dfrac{K r - \bar{\mu }}{K \Delta \epsilon _v^e} a_1 \,, \\ d_2 &= \dfrac{K r - \bar{\mu }}{K \Delta \epsilon _v^e} a_2. \end{aligned}$$

The linearization of $\eta$ comes as follows:

81 $$\begin{aligned} \begin{aligned} \dfrac{\partial \eta }{\partial \Delta \boldsymbol{\varepsilon }}&= \textbf{D}_2 + d_3 \dfrac{\partial \Delta \phi }{\partial \Delta \boldsymbol{\varepsilon }} \\&\quad+ d_4 \dfrac{\partial q}{\partial \Delta \boldsymbol{\varepsilon }} \,, \\ \textbf{D}_2&= -3 \eta ^2 \dfrac{\Delta \phi }{q} \left( g_q \textbf{D}_1 + \bar{\mu } g_{pq} \textbf{A}_1 \right) \,, \\ d_3&= -3 \eta ^2 \left( \left[ \dfrac{\Delta \phi }{q} d_1 + \dfrac{\bar{\mu }}{q} \right] g_q + \dfrac{\bar{\mu } \Delta \phi }{q} g_{pq} a_1 \right) \,, \\ d_4&= -3 \eta ^2 \dfrac{\Delta \phi }{q} \left( \left[ d_2 - \dfrac{\bar{\mu }}{q} \right] g_q + \bar{\mu } [ g_{pq} a_2 + g_{qq} ] \right) . \end{aligned} \end{aligned}$$

The linearization of $\textbf{s}$ comes as follows:

82 $$\begin{aligned} \begin{aligned} \dfrac{\partial \textbf{s}}{\partial \Delta \boldsymbol{\varepsilon }}&= 2 \eta \left( \bar{\mu } {\mathbb {I}}_d + \Delta \boldsymbol{\varepsilon }_d \otimes \dfrac{\partial \bar{\mu }}{\partial \Delta \boldsymbol{\varepsilon }} \right) \\&\quad+ \left( \textbf{s}_n + 2 \bar{\mu } \Delta \boldsymbol{\varepsilon }_d \right) \otimes \dfrac{\partial \eta }{\partial \Delta \boldsymbol{\varepsilon }} \\&= {\mathbb {D}}_1 + \textbf{D}_3 \otimes \dfrac{\partial \Delta \phi }{\partial \Delta \boldsymbol{\varepsilon }} \\&\quad+ \textbf{D}_4 \otimes \dfrac{\partial q}{\partial \Delta \boldsymbol{\varepsilon }} \,, \\ {\mathbb {D}}_1&= 2 \eta \bar{\mu } {\mathbb {I}}_d + 2 \eta \Delta \boldsymbol{\varepsilon }_d \otimes \textbf{D}_1 \\&\quad+ \left( \textbf{s}_n + 2 \bar{\mu } \Delta \boldsymbol{\varepsilon }_d \right) \otimes \textbf{D}_2 \,, \\ \textbf{D}_3&= d_3 \textbf{s}_n + 2 \left( \bar{\mu } d_3 + \eta d_1 \right) \Delta \boldsymbol{\varepsilon }_d \,, \\ \textbf{D}_4 &= d_4 \textbf{s}_n + 2 \left( \bar{\mu } d_4 + \eta d_2 \right) \Delta \boldsymbol{\varepsilon }_d. \end{aligned} \end{aligned}$$

From the relation $2 q \dfrac{\partial q}{\partial \Delta \boldsymbol{\varepsilon }} = 3 \textbf{s}: \dfrac{\partial \textbf{s}}{\partial \Delta \boldsymbol{\varepsilon }}$, the linearization of *q* reduces to:

83 $$\begin{aligned} \begin{aligned} \dfrac{\partial q}{\partial \Delta \boldsymbol{\varepsilon }}&= \textbf{D}_5 + d_5 \dfrac{\partial \Delta \phi }{\partial \Delta \boldsymbol{\varepsilon }} \,, \\ \textbf{D}_5 &= \dfrac{3}{2} \dfrac{d_6}{q} \textbf{s}: {\mathbb {D}}_1 \,, \\ d_5&= \dfrac{3}{2} \dfrac{d_6}{q} \textbf{s}: \textbf{D}_3 \,, \\ d_6 &= \left( 1 - \dfrac{3}{2} \dfrac{1}{q} \textbf{s}: \textbf{D}_4 \right) ^{-1}. \end{aligned} \end{aligned}$$

Replacing (83) back into (77), (78) and (82), we obtain the dependency of linearization of *p*, $p_c$ and $\textbf{s}$ on only $\dfrac{\partial \Delta \phi }{\partial \Delta \boldsymbol{\varepsilon }}$:

84 $$\begin{aligned} \dfrac{\partial p}{\partial \Delta \boldsymbol{\varepsilon }} &= \textbf{A} + a \dfrac{\partial \Delta \phi }{\partial \Delta \boldsymbol{\varepsilon }} \,, \\ \textbf{A} &= \textbf{A}_1 + a_2 \textbf{D}_5 \,, \\ a &= a_1 + a_2 d_5 \,, \\ \dfrac{\partial p_c}{\partial \Delta \boldsymbol{\varepsilon }} &= \textbf{B} + b \dfrac{\partial \Delta \phi }{\partial \Delta \boldsymbol{\varepsilon }} \,, \\ \textbf{B} &= \textbf{B}_1 + b_2 \textbf{D}_5 \,, \\ b &= b_1 + b_2 d_5 \,, \\ \dfrac{\partial \textbf{s}}{\partial \Delta \boldsymbol{\varepsilon }} &= {\mathbb {D}} + \textbf{D} \dfrac{\partial \Delta \phi }{\partial \Delta \boldsymbol{\varepsilon }} \,, \\ {\mathbb {D}} &= {\mathbb {D}}_1 + \textbf{D}_4 \otimes \textbf{D}_5 \,, \\ \textbf{D} &= \textbf{D}_3 + d_5 \textbf{D}_4. \end{aligned}$$

### F. Linearization of the sub-stepping scheme, CASM model

Taking linearization of (9) for the sub-step $(k+1)$, with the remark that linearization of ${}^k p$ does not vanish, one obtains:

85 $$\begin{aligned} \dfrac{\partial \, {}^{k+1}p}{\partial \Delta \boldsymbol{\varepsilon }} &= \dfrac{{}^{k+1}p}{{}^{k}p} \dfrac{\partial \, {}^{k}p}{\partial \Delta \boldsymbol{\varepsilon }} \\&\quad+ {}^{k+1} K \dfrac{\partial \, {}^{k+1}\Delta \varepsilon ^e_v}{\partial \Delta \boldsymbol{\varepsilon }} \,, \\ {}^{k+1} K &= \dfrac{1+e}{\kappa } {}^{k+1} p. \end{aligned}$$

It is noted that ${}^{k+1} \Delta \varepsilon ^e_v = {}^{k+1} \Delta \epsilon _v - \Delta \phi g_p$, hence:

86 $$\begin{aligned} \dfrac{\partial \, {}^{k+1} \Delta \varepsilon ^e_v}{\partial \Delta \boldsymbol{\varepsilon }} &= \alpha _{k+1} \textbf{I} \\&\quad- \Delta \phi \left( g_{pp} \dfrac{\partial \, {}^{k+1} p}{\partial \Delta \boldsymbol{\varepsilon }} + g_{pq} \dfrac{\partial \, {}^{k+1} q}{\partial \Delta \boldsymbol{\varepsilon }} \right) \\&\quad- g_p \dfrac{\partial \Delta \phi }{\partial \Delta \boldsymbol{\varepsilon }}. \end{aligned}$$

In Eq. (86) and further equations below, $g_p$, $g_q$, $g_{pp}$, $g_{pq}$ and $g_{qq}$ are evaluated at $({}^{k+1} p, {}^{k+1} q)$.

From (85) and (86), one can rewrite $\dfrac{\partial \, {}^{k+1} p}{\partial \Delta \boldsymbol{\varepsilon }}$ as dependent term of $\dfrac{\partial \, {}^{k+1} q}{\partial \Delta \boldsymbol{\varepsilon }}$ and $\dfrac{\partial \Delta \phi }{\partial \Delta \boldsymbol{\varepsilon }}$:

87 $$\begin{aligned} \dfrac{\partial \, {}^{k+1} p}{\partial \Delta \boldsymbol{\varepsilon }} &= \textbf{A}_1 + a_1 \dfrac{\partial \Delta \phi }{\partial \Delta \boldsymbol{\varepsilon }} \\&\quad+ a_2 \dfrac{\partial \, {}^{k+1} q}{\partial \Delta \boldsymbol{\varepsilon }} \,, \\ \textbf{A}_1 &= a_3 \left( \dfrac{{}^{k+1}p}{{}^{k}p} \dfrac{\partial \, {}^{k}p}{\partial \Delta \boldsymbol{\varepsilon }} + \alpha _{k+1} \, {}^{k+1} K \textbf{I} \right) \,, \\ a_1 &= -a_3 {}^{k+1} K g_p \,, \quad a_2 = -a_3 {}^{k+1} K \Delta \phi g_{pq} \,, \\ a_3 &= \left( 1 + {}^{k+1} K \Delta \phi g_{pp} \right) ^{-1}. \end{aligned}$$

Taking the linearization of (11):

88 $$\begin{aligned} \begin{aligned} \dfrac{\partial \, {}^{k+1} p_c}{\partial \Delta \boldsymbol{\varepsilon }}&= \dfrac{{}^{k+1} p_c}{{}^{k} p_c} \dfrac{\partial \, {}^{k} p_c}{\partial \Delta \boldsymbol{\varepsilon }} \\&\quad+ \theta \, {}^{k+1} p_c \left( \alpha _{k+1} \textbf{I} - \dfrac{\partial \, {}^{k+1} \Delta \varepsilon ^e_v}{\partial \Delta \boldsymbol{\varepsilon }} \right) \\&= \textbf{B}_1 + b_1 \dfrac{\partial \Delta \phi }{\partial \Delta \boldsymbol{\varepsilon }} + b_2 \dfrac{\partial \, {}^{k+1} q}{\partial \Delta \boldsymbol{\varepsilon }} \,, \\ \textbf{B}_1&= \dfrac{{}^{k+1} p_c}{{}^{k} p_c} \dfrac{\partial \, {}^{k} p_c}{\partial \Delta \boldsymbol{\varepsilon }} \\&\quad+ \dfrac{\theta \, {}^{k+1} p_c}{{}^{k+1} K} \left( \alpha _{k+1} {}^{k+1} K \textbf{I} + \dfrac{{}^{k+1} p}{{}^{k} p} \dfrac{\partial \, {}^{k} p}{\partial \Delta \boldsymbol{\varepsilon }} - \textbf{A}_1 \right) \,, \\ b_1&= -\dfrac{\theta \, {}^{k+1} p_c}{{}^{k+1} K} a_1 \,, \quad b_2 = -\dfrac{\theta \, {}^{k+1} p_c}{{}^{k+1} K} a_2. \end{aligned} \end{aligned}$$

The deviatoric pressure reads:

89 $$\begin{aligned} {}^{k+1} \textbf{s} &= \eta \left( {}^k \textbf{s} + 2 \bar{\mu } \, {}^{k+1}\Delta \boldsymbol{\varepsilon }_d \right) \,, \\ \bar{\mu } &= \dfrac{{}^{k+1} p - {}^k p}{{}^{k+1} \, \Delta \epsilon _v^e} r \,, \\ \eta &= \left( 1 + 3 \bar{\mu } \dfrac{\Delta \phi }{{}^{k+1} q} g_q \right) ^{-1}. \end{aligned}$$

The linearization of $\bar{\mu }$ comes as follows:

90 $$\begin{aligned} \begin{aligned} \dfrac{\partial \bar{\mu }}{\partial \Delta \boldsymbol{\varepsilon }}&= \textbf{D}_1 + d_1 \dfrac{\partial \Delta \phi }{\partial \Delta \boldsymbol{\varepsilon }} \\&\quad+ d_2 \dfrac{\partial \, {}^{k+1} q}{\partial \Delta \boldsymbol{\varepsilon }} \,, \\ \textbf{D}_1&= \dfrac{{}^{k+1} K r - \bar{\mu }}{{}^{k+1} K \, {}^{k+1} \Delta \epsilon _v^e} \textbf{A}_1 \\&\quad- \dfrac{1}{^{k+1} \Delta \epsilon _v^e} \left( r - \dfrac{\bar{\mu }}{^{k+1} K} \dfrac{^{k+1} p}{^{k} p} \right) \dfrac{\partial \, ^{k} p}{\partial \Delta \boldsymbol{\varepsilon }} \,, \\ d_1&= \dfrac{{}^{k+1} K r - \bar{\mu }}{{}^{k+1} K \, {}^{k+1} \Delta \epsilon _v^e} a_1 \,, \qquad d_2 = \dfrac{{}^{k+1} K r - \bar{\mu }}{{}^{k+1} K \, {}^{k+1} \Delta \epsilon _v^e} a_2. \end{aligned} \end{aligned}$$

The linearization of $\eta$ comes as follows:

91 $$\begin{aligned} \begin{aligned} \dfrac{\partial \eta }{\partial \Delta \boldsymbol{\varepsilon }}&= \textbf{D}_2 + d_3 \dfrac{\partial \Delta \phi }{\partial \Delta \boldsymbol{\varepsilon }} \\&\quad+ d_4 \dfrac{\partial \, {}^{k+1} q}{\partial \Delta \boldsymbol{\varepsilon }} \,, \\ \textbf{D}_2&= -3 \eta ^2 \dfrac{\Delta \phi }{{}^{k+1} q} \left( g_q \textbf{D}_1 + \bar{\mu } g_{pq} \textbf{A}_1 \right) \,, \\ d_3&= -3 \eta ^2 \left( \left[ \dfrac{\Delta \phi }{{}^{k+1} q} d_1 + \dfrac{\bar{\mu }}{{}^{k+1} q} \right] g_q + \dfrac{\bar{\mu } \Delta \phi }{{}^{k+1} q} g_{pq} a_1 \right) \,, \\ d_4&= -3 \eta ^2 \dfrac{\Delta \phi }{{}^{k+1} q} \left( \left[ d_2 - \dfrac{\bar{\mu }}{{}^{k+1} q} \right] g_q + \bar{\mu } [ g_{pq} a_2 + g_{qq} ] \right) . \end{aligned} \end{aligned}$$

The linearization of ${}^{k+1} \textbf{s}$ comes as follows:

92 $$\begin{aligned} \begin{aligned} \dfrac{\partial \, {}^{k+1} \textbf{s}}{\partial \Delta \boldsymbol{\varepsilon }}&= \eta \left( \dfrac{\partial \, {}^{k} \textbf{s}}{\partial \Delta \boldsymbol{\varepsilon }} + 2 \alpha _{k+1} \bar{\mu } {\mathbb {I}}_d + 2 \, {}^{k+1} \Delta \boldsymbol{\varepsilon }_d \otimes \dfrac{\partial \bar{\mu }}{\partial \Delta \boldsymbol{\varepsilon }} \right) \\&\quad+ \left( {}^{k} \textbf{s} + 2 \bar{\mu } \, {}^{k+1} \Delta \boldsymbol{\varepsilon }_d \right) \otimes \dfrac{\partial \eta }{\partial \Delta \boldsymbol{\varepsilon }} \\&= {\mathbb {D}}_1 + \textbf{D}_3 \otimes \dfrac{\partial \Delta \phi }{\partial \Delta \boldsymbol{\varepsilon }} + \textbf{D}_4 \otimes \dfrac{\partial \, {}^{k+1} q}{\partial \Delta \boldsymbol{\varepsilon }} \,, \\ {\mathbb {D}}_1&= \eta \left( \dfrac{\partial \, {}^{k} \textbf{s}}{\partial \Delta \boldsymbol{\varepsilon }} + 2 \, \alpha _{k+1} \bar{\mu } {\mathbb {I}}_d + 2 \, {}^{k+1} \Delta \boldsymbol{\varepsilon }_d \otimes \textbf{D}_1 \right) \\&\quad+ \left( {}^{k} \textbf{s} + 2 \bar{\mu } \, {}^{k+1} \Delta \boldsymbol{\varepsilon }_d \right) \otimes \textbf{D}_2 \,, \\ \textbf{D}_3&= d_3 \, {}^k \textbf{s} + 2 \left( \bar{\mu } d_3 + \eta d_1 \right) \, {}^{k+1} \Delta \boldsymbol{\varepsilon }_d \,, \\ \textbf{D}_4 &= d_4 \, {}^k \textbf{s} + 2 \left( \bar{\mu } d_4 + \eta d_2 \right) \, {}^{k+1} \Delta \boldsymbol{\varepsilon }_d. \end{aligned} \end{aligned}$$

From the relation $2 \, {}^{k+1} q \dfrac{\partial \, {}^{k+1} q}{\partial \Delta \boldsymbol{\varepsilon }} = 3 \, {}^{k+1} \textbf{s}: \dfrac{\partial \, {}^{k+1} \textbf{s}}{\partial \Delta \boldsymbol{\varepsilon }}$, the linearization of ${}^{k+1} q$ reduces to:

93 $$\begin{aligned} \begin{aligned}\dfrac{\partial \, {}^{k+1} q}{\partial \Delta \boldsymbol{\varepsilon }} &= \textbf{D}_5 + d_5 \dfrac{\partial \Delta \phi }{\partial \Delta \boldsymbol{\varepsilon }} \,, \\ \textbf{D}_5 &= \dfrac{3}{2} \dfrac{d_6}{{}^{k+1} q} {}^{k+1} \textbf{s}: {\mathbb {D}}_1 \,, \\d_5 &= \dfrac{3}{2} \dfrac{d_6}{{}^{k+1} q} {}^{k+1} \textbf{s}: \textbf{D}_3 \,, \\ d_6 &= \left( 1 - \dfrac{3}{2} \dfrac{1}{{}^{k+1} q} {}^{k+1} \textbf{s}: \textbf{D}_4 \right) ^{-1}. \end{aligned} \end{aligned}$$

Replacing (93) back into (87), (88) and (92), we obtain the dependency of linearization of *p*, $p_c$ and $\textbf{s}$ on only $\dfrac{\partial \Delta \phi }{\partial \Delta \boldsymbol{\varepsilon }}$:

94 $$\begin{aligned} \dfrac{\partial \, {}^{k+1} p}{\partial \Delta \boldsymbol{\varepsilon }} &= \textbf{A} + a \dfrac{\partial \Delta \phi }{\partial \Delta \boldsymbol{\varepsilon }} \,, \\ \textbf{A} &= \textbf{A}_1 + a_2 \textbf{D}_5 \,, \quad a = a_1 + a_2 d_5 \,, \\ \dfrac{\partial \, {}^{k+1} p_c}{\partial \Delta \boldsymbol{\varepsilon }} &= \textbf{B} + b \dfrac{\partial \Delta \phi }{\partial \Delta \boldsymbol{\varepsilon }} \,, \\ \textbf{B} &= \textbf{B}_1 + b_2 \textbf{D}_5 \,, \quad b = b_1 + b_2 d_5 \,, \\ \dfrac{\partial \, {}^{k+1} \textbf{s}}{\partial \Delta \boldsymbol{\varepsilon }} &= {\mathbb {D}} + \textbf{D} \dfrac{\partial \Delta \phi }{\partial \Delta \boldsymbol{\varepsilon }} \,, \\ {\mathbb {D}} &= {\mathbb {D}}_1 + \textbf{D}_4 \otimes \textbf{D}_5 \,, \\ \textbf{D} &= \textbf{D}_3 + d_5 \textbf{D}_4. \end{aligned}$$

### G. Analytical solution of CASM model subjected to drained proportional stress path

In the proportional loading scheme, the loading is governed by:

95 $$\begin{aligned} \dot{q} = k \dot{p} \,, \qquad k = const. \end{aligned}$$

For further derivation, we denote $\beta = \dfrac{q}{p}$, subsequently:

96 $$\begin{aligned} q = \beta p \,, \qquad \dot{q} = \dot{\beta } p + \beta \dot{p}. \end{aligned}$$

Because of Eq. (95), one can deduce

97 $$\begin{aligned} k \dot{p} = \dot{\beta } p + \beta \dot{p} \qquad \Rightarrow \qquad \dfrac{\dot{p}}{p} = \dfrac{\dot{\beta }}{k - \beta }. \end{aligned}$$

#### Solution for volumetric strain

##### Elastic domain

The Eqs. (2) and (3) leads to:

98 $$\begin{aligned} \dot{\varepsilon }^e_v = \dfrac{\kappa }{1 + e} \dfrac{\dot{p}}{p}. \end{aligned}$$

In the elastic domain: $\dot{\varepsilon }_v = \dot{\varepsilon }^e_v$. Hence, $\Delta \epsilon _v = \Delta \varepsilon ^e_v$ can be approximated by:

99 $$\begin{aligned} \Delta \varepsilon ^e_v = \dfrac{\kappa }{1 + e} \Delta \ln p = \dfrac{\kappa }{1 + e} \ln \dfrac{p_{n+1}}{p_n}. \end{aligned}$$

##### Plastic domain

In the plastic domain, $\Delta \varepsilon ^e_v$ can be calculated using Eq. (99). To compute $\Delta {\varepsilon }^p_v$, we start by taking the time derivative of yield function (6):

100 $$\begin{aligned} \dfrac{\dot{p}_c}{p_c} = \dfrac{\dot{p}}{p} + N \ln R \dfrac{q^{N-1} \dot{q}}{(M p)^N} - N \ln R \, \dfrac{\dot{p}}{p} \left( \dfrac{q}{M p} \right) ^N. \end{aligned}$$

Taking into account Eqs. (95) and (97), Eq. (100) can be rewritten as:

101 $$\begin{aligned} \dfrac{\dot{p}_c}{p_c} &= \dfrac{\dot{p}}{p} \left[ 1 + \dfrac{k N \ln R}{M^N} \beta ^{N-1} - \dfrac{N \ln R}{M^N} \beta ^N \right] \\&= \dfrac{\dot{\beta }}{k - \beta } + \dfrac{k N \ln R}{M^N} \dfrac{\beta ^{N-1} \dot{\beta }}{k - \beta } - \dfrac{N \ln R}{M^N} \dfrac{\beta ^N \dot{\beta }}{k - \beta }. \end{aligned}$$

From the hardening rule (4), $\Delta \varepsilon ^p_v$ can be computed as:

102 $$\begin{aligned} \Delta \varepsilon ^p_v &= \dfrac{1}{\theta } \biggl [ - \ln (k-\beta ) |_{\beta _n}^{\beta _{n+1}} + \dfrac{k N \ln R}{M^N}\\ &\quad \int _{\beta _n}^{\beta _{n+1}} \dfrac{\beta ^{N-1}}{k - \beta } d \beta - \dfrac{N \ln R}{M^N} \int _{\beta _n}^{\beta _{n+1}} \dfrac{\beta ^N}{k - \beta } d \beta \biggr ]. \end{aligned}$$

Denote $F_N(x)$ as the primitive integral of $\dfrac{x^N}{1-x}$, such that $F_N'(x) = \dfrac{x^N}{1-x}$, Eq. (102) can be rewritten as:

103 $$\begin{aligned} \Delta \varepsilon ^p_v = \dfrac{1}{\theta } \left( - \ln (k-\beta ) + \dfrac{k^N N \ln R}{M^N} \left[ F_{N-1} \left( \dfrac{\beta }{k} \right) - F_{N} \left( \dfrac{\beta }{k} \right) \right] \right) \bigg |_{\beta _n}^{\beta _{n+1}}. \end{aligned}$$

It is noted that, in the case that *N* is a positive integer, $F_N(x)$ can be determined recursively as following:

104 $$\begin{aligned} F_0(x) = -\ln (1-x) \,, \qquad F_{N+1}(x) = F_N(x) - \dfrac{x^{N+1}}{N+1}. \end{aligned}$$

#### Solution for deviatoric strain

##### Elastic domain

The rate of elastic shear strain follows by $\dot{\varepsilon }^e_q = \dfrac{\dot{q}}{3 \mu }$, hence:

105 $$\begin{aligned} \dot{\varepsilon }^e_q = \dfrac{\kappa k}{3 r (1+e)} \dfrac{\dot{p}}{p}. \end{aligned}$$

In the elastic domain: $\dot{\varepsilon }_q = \dot{\varepsilon }^e_q$. Therefore, $\Delta \epsilon _q = \Delta \varepsilon ^e_q$ can be approximated by:

106 $$\begin{aligned} \Delta \epsilon _q = \dfrac{\kappa k}{3 r (1 + e)} \Delta \left( \ln p \right) = \dfrac{\kappa k}{3 r (1 + e)} \ln \dfrac{p_{n+1}}{p_n}. \end{aligned}$$

##### Plastic domain

In the plastic domain, $\Delta \varepsilon ^e_q$ can be calculated using Eq. (106). To compute $\Delta \varepsilon 0_q$, we start first by the assumption:

107 $$\begin{aligned} \dot{\varepsilon }^p_q \dfrac{\partial g}{\partial p} = \dot{\varepsilon }^p_v \dfrac{\partial g}{\partial q} \Leftrightarrow \dot{\varepsilon }^p_q = \dot{\varepsilon }^p_v \, \dfrac{3(3 + M) - 2M \beta }{9(M - \beta )}. \end{aligned}$$

From Eqs. (107 and (101), we come up with the equation for $\dot{\varepsilon }^p_q$ which only depends on $\beta$ and $\dot{\beta }$:

108 $$\begin{aligned} \begin{aligned} \dot{\varepsilon }^p_q = \dfrac{1}{\theta } \dfrac{3(3 + M) - 2M \beta }{9(M - \beta )} \left( \dfrac{\dot{\beta }}{k - \beta } + \dfrac{k N \ln R}{M^N} \dfrac{\beta ^{N-1} \dot{\beta }}{k - \beta } - \dfrac{N \ln R}{M^N} \dfrac{\beta ^N \dot{\beta }}{k - \beta } \right) . \end{aligned} \end{aligned}$$

Using the relation:

109 $$\begin{aligned} \begin{aligned}\dfrac{3(3+M) - 2M \beta }{(M - \beta )(k - \beta )} &= \dfrac{A}{M - \beta } + \dfrac{B}{k - \beta } \,, \\ A &= \dfrac{2M^2 - 3(3+M)}{M-k} \,, \\B &= \dfrac{3(3+M) - 2kM}{M-k}. \end{aligned} \end{aligned}$$

Then the Eq. (108) can be expanded as:

110 $$\begin{aligned} \begin{aligned} \dot{\varepsilon }^p_q&= \dfrac{A}{9 \theta } \left( \dfrac{\dot{\beta }}{M - \beta } + \dfrac{k N \ln R}{M^N} \dfrac{\beta ^{N-1} \dot{\beta }}{M - \beta } - \dfrac{N \ln R}{M^N} \dfrac{\beta ^N \dot{\beta }}{M - \beta } \right) \\&\quad+ \dfrac{B}{9 \theta } \left( \dfrac{\dot{\beta }}{k - \beta } + \dfrac{k N \ln R}{M^N} \dfrac{\beta ^{N-1} \dot{\beta }}{k - \beta } - \dfrac{N \ln R}{M^N} \dfrac{\beta ^N \dot{\beta }}{k - \beta } \right) \\&= \dfrac{A}{9 \theta } \left( \dfrac{\dot{\beta }}{M - \beta } + \dfrac{k N \ln R}{M^N} \dfrac{\beta ^{N-1} \dot{\beta }}{M - \beta } - \dfrac{N \ln R}{M^N} \dfrac{\beta ^N \dot{\beta }}{M - \beta } \right) + \dfrac{B}{9} \dot{\varepsilon }^p_v. \end{aligned} \end{aligned}$$

Which leads to the equation of $\Delta \varepsilon ^p_q$:

111 $$\begin{aligned} \Delta \varepsilon ^p_q &= \dfrac{A}{9 \theta } \left( -\ln (M-\beta ) + N \ln R \left[ \dfrac{k}{M} F_{N-1} \left( \dfrac{\beta }{M} \right) - F_N \left( \dfrac{\beta }{M} \right) \right] \right) \bigg |_{\beta _n}^{\beta _{n+1}} \\&\quad+ \dfrac{B}{9} \Delta \varepsilon ^p_v. \end{aligned}$$

## List of symbols

$$\alpha $$: Load increment
$$\bar{\mu }$$: Shear stiffness
$$\boldsymbol{\sigma }$$: Stress tensor
$$\boldsymbol{\varepsilon }$$: Strain tensor
$$\kappa $$: Swelling index
$$\lambda $$: Compression index
$$\mathbb {C}$$: Tangent operator
$\mathbb {I}_d$: Symmetric deviatoric tensor
$$\textbf{I}$$: Identity matrix
$$\textbf{s}$$: Deviatoric stress
$$\nu $$: Poisson ratio
$$\phi $$: Plastic multiplier
$$\rho $$: Density
*e*: Void ratio
*K*: Bulk modulus
*M*: Slope of critical state line
*N*: Shape parameter
*p*: Hydrostatic pressure
$$p_c$$: Preconsolidation pressure
*q*: Deviatoric invariant
*R*: Spacing ratio
